# Spontaneous Formation of a Sustainable Antifreeze Coating by Peptide Self-Assembly

**DOI:** 10.1021/acsami.4c22816

**Published:** 2025-03-02

**Authors:** Michaela Kaganovich, Eilam Gibeon, Anna Shilling Bakalinsky, Deborah E. Shalev, Ido Braslavsky, Meital Reches

**Affiliations:** † Institute of Chemistry, The Hebrew University of Jerusalem, Jerusalem 9190401, Israel; ‡ The Center for Nanoscience and Nanotechnology, The Hebrew University of Jerusalem, Jerusalem 9190401, Israel; § Wolfson Centre for Applied Structural Biology, The Hebrew University of Jerusalem, Jerusalem 9190500, Israel; ∥ Department of Pharmaceutical Engineering, Azrieli College of Engineering, Jerusalem 9103501, Israel; ⊥ The Robert H. Smith Faculty of Agriculture, Food and Environment, Institute of Biochemistry, Food Science, and Nutrition, The Hebrew University of Jerusalem, Rehovot 7610001, Israel

**Keywords:** peptide, self-assembly, antifreeze, coating, ice-binding

## Abstract

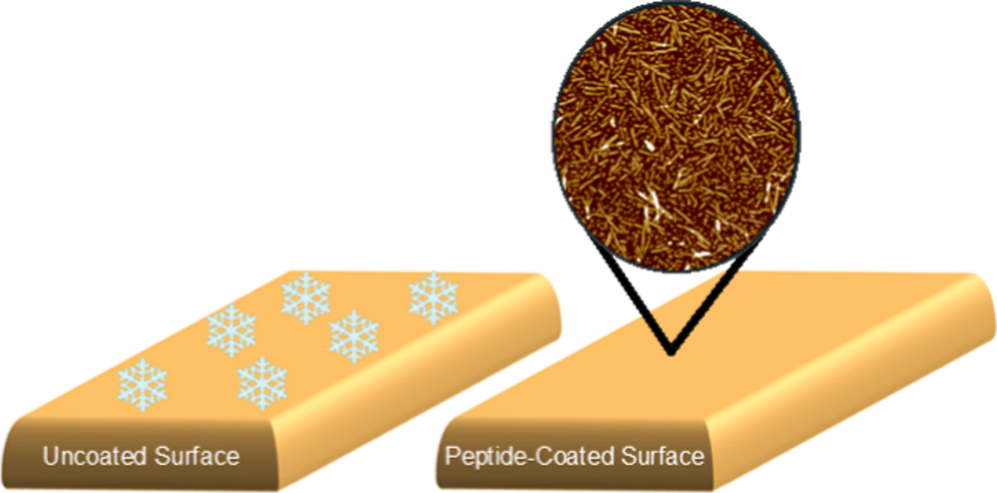

The formation of ice and frost on surfaces poses significant challenges to aviation, crop protection, organ preservation, and other fields. This paper presents the formation of sustainable antifreeze coating by the self-assembly of short peptides. The peptide design is inspired by and combines different elements from distinct natural proteins: (i) a sequence of amino acids from an antifreeze protein and (ii) the amino acids 3,4-dihydroxyphenylalanine (DOPA) and lysine from mussel adhesion proteins that anchor the peptide to a surface. The peptide, termed AFPep1, incorporates the repetitive ice-binding motif found in the antifreeze protein of the longhorn beetle (*Rhagium inquisitor*). Surfaces coated with the peptide exhibited antifreeze activity with a delay of the initial freezing of 5 °C degrees compared to a bare surface. Furthermore, AFPep1 exhibited relatively effective ice recrystallization inhibition (IRI) activity in solution compared to various other common substances, with an inhibition concentration of 0.5 ± 0.1 mM. Additionally, the presence of AFPep1 in the solution shaped ice crystals into hexagonal plates, indicating specific binding to ice. Moreover, thermal hysteresis results show that AFPep1 completely inhibits ice growth at supercooling levels of up to 0.04 °C at 2 mM, indicating the peptide’s ability to self-assemble and create high-density anchoring points on the ice surface. These results highlight the significant potential of specific peptides as antifreeze coatings for technological infrastructure and agricultural applications.

## Introduction

Global warming has intensified the frequency and severity of extreme weather events, with a notable increase in massive snowstorms and record-low temperatures, presenting challenges beyond daily life and impacting both natural and technological infrastructure. Ice and frost formation on surfaces, such as heat exchangers, insulators, and aircraft wings, poses significant threats to efficiency, safety, and overall performance.^1,2^ The consequences of these cold conditions extend to agriculture, with frost accumulation on plant surfaces causing crop injuries and agricultural disasters.^3^

Numerous efforts are being invested to advance the development of coatings with anti-ice and antifreeze properties. For instance, using superhydrophobic coatings reduces surface energy, consequently mitigating the adherence of water droplets to the surface.^4^ However, these coatings typically exhibit instability where icing, deicing, and high humidity occur.^4,5^ Another approach to anti-ice coatings involves applying aqueous lubricating coatings, employing polymers with hydrophilic groups. This strategy effectively reduces ice adhesion by creating a smoother surface and exploiting water’s relatively low freezing point within the lubricating layer compared to bulk water.^6^ Moreover, various materials, such as polydimethylsiloxane,^7^ fluorosilane-modified epoxy,^8^ and fluorine–silicone resin,^9^ are employed for surface modification to confer anti-ice properties. However, many of these materials involve prolonged application processes, are not environmentally friendly, and have high production costs.

Another promising material to mitigate the accumulation of ice and frost on surfaces involves utilizing antifreeze proteins (AFP).^10^ These proteins are naturally produced by various organisms, such as fish, insects, and fungi, enabling the organisms to endure subzero conditions. AFPs exhibit diverse secondary structures, such as α-helices, β-sheets, and β-helices, individually or in combination, with sizes ranging from approximately 3 to 33 kDa.^11^ Furthermore, these proteins typically incorporate diverse amino acid sequences that actively contribute to their functionality.^12^ AFPs effectively control the growth and recrystallization of ice crystals by adsorbing to the front of the ice crystal plane, impeding the crystal’s growth and decreasing the freezing process. Importantly, these proteins can depress the freezing point of water below the equilibrium melting point, thereby restricting ice growth for an extended duration.^13^

Acquiring sufficient amounts of AFP for this purpose is a challenge. It is achievable through either extraction from living organisms or by employing recombinant expression in bacteria, a relatively time-consuming process that involves multiple purification steps.^14^ Attaching AFPs to the desired surface requires various strategies and involves multiple steps. Previous studies have shown diverse methods for surface attachment of AFPs, including polymers and modified AFPs. For instance, adding a ketone group to AFPs facilitates their attachment to polymer chains on a glass surface.^15^ Alternatively, a modification enabling ionic attachment to aluminum surfaces treated with plasma has been explored.^16^ Another study utilized a peptide as a linker between AFPs and the aluminum surface.^17^ Overall, the production and attachment of AFPs to surfaces present complicated challenges, suggesting the need for innovative methods to simplify these processes.

Peptides are promising candidates as antifreeze and anti-ice agents due to their wide-ranging efficacy, biocompatibility, and low toxicity.^18−21^ Synthesizing short peptides with sequences derived from the active antifreeze motif of the AFP emerges as a good solution. This approach is relatively straightforward in synthesis and purification, allows for high-volume production, and provides the flexibility to modify the sequence for diverse functionalities. In earlier research, peptides synthesized with amino-acid sequences identical to partial sequences of winter flounder fish antifreeze were explored for surface applications and attached to a polydopamine-coated silicon wafer.^22^ This investigation demonstrated that the modified surface effectively lowered the freezing temperature of water droplets. Another study found that a glass surface coated with a silane coupling agent could reduce the supercooling temperature and the adhesion strength of frozen droplets.^23^

Here, we present a peptide sequence that is derived from AFPs. The peptide sequence is derived from a repetitive ice-binding motif found in the antifreeze protein of the Longhorn beetle, *Rhagium inquisitor*. *R. inquisitor* has a wide distribution throughout Europe and in the frigid regions of Siberia. It expresses AFPs in its tissues and hemolymph, enabling it to endure temperatures below −25 °C,^24,25^ with thermal hysteresis estimated to approach nearly 6.5 °C, making it one of the highest among all species.^24^ This AFP has a β-solenoid structure forming a sandwich with six- and seven-stranded sheets cross-linked by cysteine residues, creating a disulfide bridge. The ice-binding surface of this protein features the TxT motif, where threonine residues are spaced to mimic the arrangement of oxygen atoms in ice.^26^ This arrangement perfectly matches the planes of ice, thereby reducing the temperature required for ice crystal growth. Previously, various short peptide sequences were derived from distinct positions in this protein’s ice-binding motif sequence. Some of these peptides demonstrated antifreeze activity by inhibiting ice crystal growth and reshaping the crystals, which indicates a high affinity of the peptides for ice planes. They also exhibited thermal hysteresis between 0.03 and 0.10 °C at a concentration of 10 mM.^27^

We have designed a peptide, inspired by a distinct anti-ice motif derived from the proteins found in *R. inquisitor*. This peptide incorporates four elements to facilitate surface attachment and self-assembly of the peptide into a coating. The first component, *L*-3,4-dihydroxyphenylalanine (DOPA), abundant in the mussel adhesive proteins,^28,29^ plays a crucial role in the attachment of the peptide to various surfaces.^30−32^ The second element, lysine, also found in mussel adhesive proteins alongside DOPA, exhibits a synergistic effect on binding DOPA to surfaces, particularly when positioned adjacent to DOPA.^33−35^ The third element, diphenylalanine enhances the self-assembly of the peptide through π–π interactions.^36,37^ Lastly, the peptide includes an anti-ice motif derived from the *R. inquisitor* protein, which confers its ice-freeze functionality.^24,25^

In this study, we present a short peptide comprising 14 amino acids strategically designed to possess three key elements capable of spontaneous self-assembly into a coating that exhibits antifreeze properties. The peptide coating demonstrated strong adherence to the surface and effective antifreeze activity.

## Results and Discussion

### Design and Synthesis of the Peptide

We designed a peptide to achieve antifreeze activity, both in solution and as a surface coating (Table 1). The peptide includes four elements: (I) the amino acid DOPA that enables the attachment to the surface, (II) the amino acid lysine that further promotes the attachment of the peptide to the surface and improves the solubility of AFPep1, (III) diphenylalanine to allow the self-assembly of the peptides into a coating, and (IV) an anti-ice motif derived from the protein of *R. inquisitor* (marked in blue, Table 1). Control peptides (AFPepC1- AFPepC6) were also investigated to evaluate how these four elements affect coating adhesion and antifreeze activity (Table 1).

**Table 1:** Sequences of the Designed Peptides

peptide	sequence	Mw (g/mol)
AFPep1	NH_2_-Lys-DOPA-Phe-Phe-Pro-Thr-Gln-Thr-Gln-Thr-Ile-Thr-Gly-Pro-CONH_2_	1645
AFPepC1	NH_2_-Lys-DOPA-Pro-Thr-Gln-Thr-Gln-Thr-Ile-Thr-Gly-Pro-CONH_2_	1351
AFPepC2	NH_2_-DOPA-Phe-Phe-Pro-Thr-Gln-Thr-Gln-Thr-Ile-Thr-Gly-Pro-CONH_2_	1518
AFPepC3	NH_2_-Phe-Phe-Pro-Thr-Gln-Thr-Gln-Thr-Ile-Thr-Gly-Pro-CONH_2_	1337
AFPepC4	NH_2_-Lys-DOPA-Phe-Phe-Pro-Ala-Gln-Ala-Gln-Ala-Ile-Ala-Gly-Pro-CONH_2_	1526
AFPepC5	NH_2_-DOPA-Phe-Phe-Pro-Ala-Gln-Ala-Gln-Ala-Ile-Ala-Gly-Pro-CONH_2_	1397
AFPepC6	NH_2_-DOPA-Phe-Phe-OMe	506

**Scheme 1:**
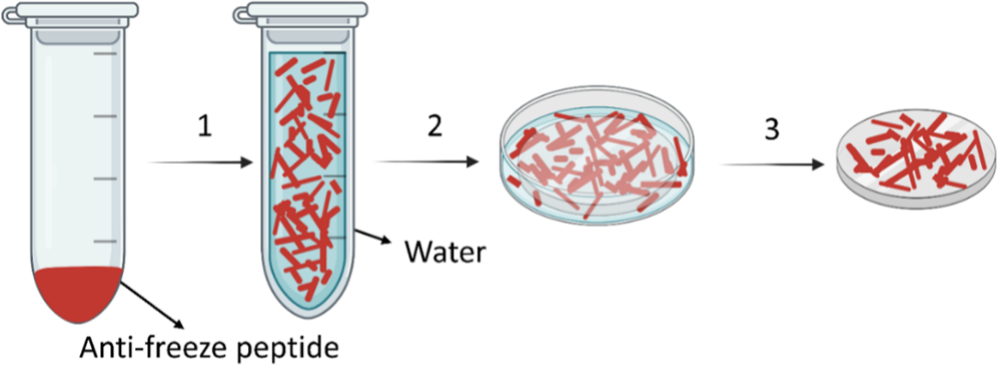
Schematic Illustration of the Antifreeze Peptide Coating Preparation Initially, the peptide, denoted by red color, was added into the tube, followed by the addition of water. Subsequently, the peptide solution was vortexed and sonicated to enhance its solubility (step 1). The silicon surface, depicted by its gray surface, was then submerged in the peptide solution overnight (step 2). Following this immersion period, the surface was rinsed with LC/MS water and dried using nitrogen gas (step 3).

Peptide coating preparation. A silicon surface was coated with peptide AFPep1 at different concentrations (5, 1, 0.2, and 0.1 mM) to optimize the antifreeze activity (Scheme 1).

### Peptide Characterization

**Figure 1:**
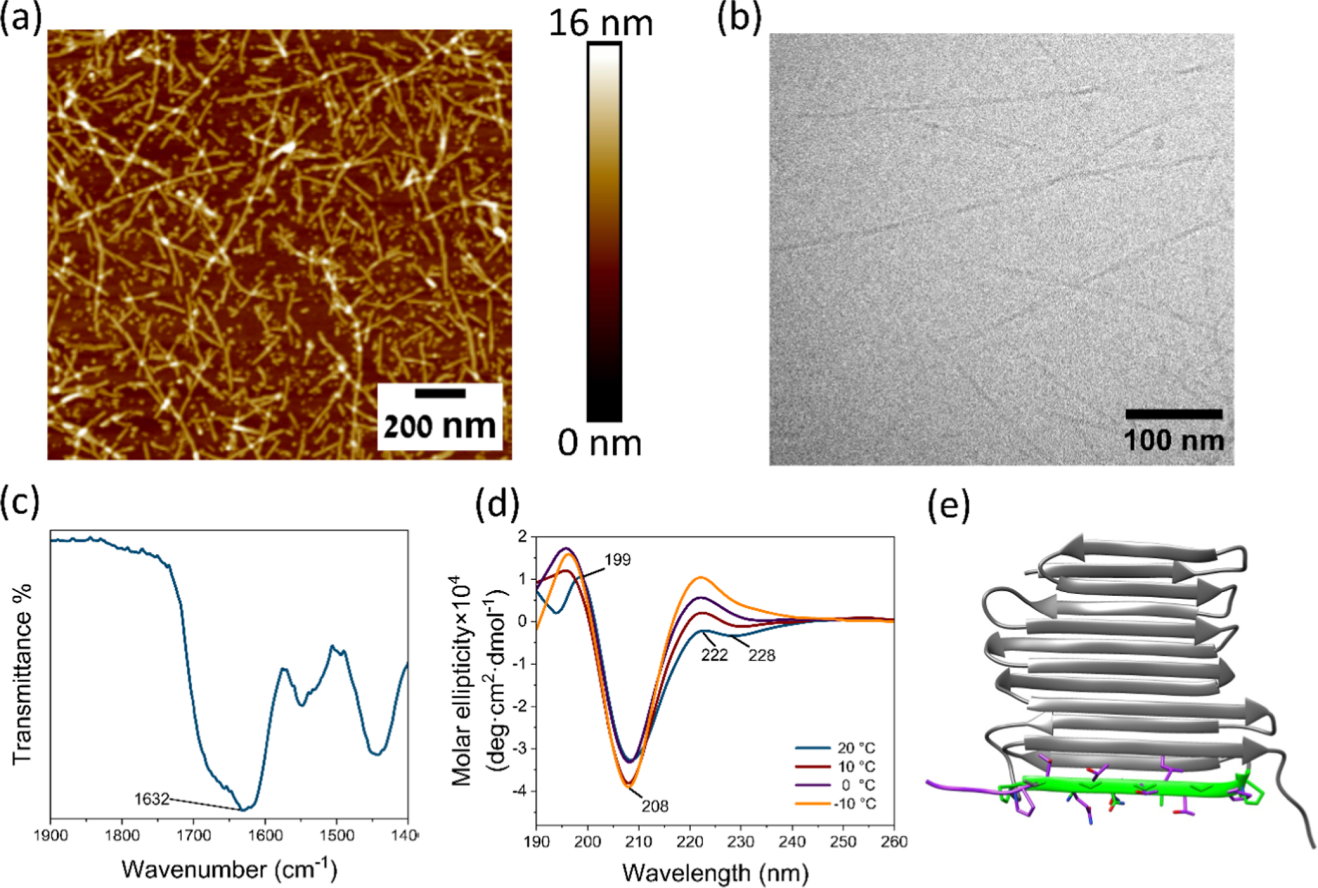
Morphological and secondary structure analysis of AFPep1 assemblies. (a) Representative AFM image of AFPep1 at a concentration of 5 mM. (b) Representative Cryo-TEM image of AFPep1 at a concentration of 5 mM. (c) FT-IR spectra. (d) CD spectra of the peptide at different temperatures. (e) AlphaFold2 calculated structures of AFPep1 (purple) overlaid on the crystal structure of *Rhagium inquisitor* antifreeze protein (PDBid 4dt5, gray and green).

Atomic force microscope (AFM) analysis revealed that at a concentration of 5 mM, AFPep1 formed thin fibrils ranging in size from 10 to 900 nm (Figure 1a). The length of the fibrils decreased with the peptide concentration (Figure S1a–c).

Cryo-transmission electron microscopy (Cryo-TEM) was utilized to analyze the assembled structures of AFPep1 in solution. The peptide was dissolved at a concentration of 5 mM and exhibited the same morphology as observed by AFM on the surface (Figure 1b). It is noteworthy that these structures appeared to be significantly longer in the solution phase.

Fourier-transform infrared spectroscopy (FT-IR) and circular dichroism (CD) were employed to assess the secondary structure of the peptides (Figure 1c,d). The FT-IR spectra of AFPep1 exhibited a peak at 1632 cm^–1^ (Figure 1c) associated with a β-sheet structure typically found within the region 1620–1640 cm^–1^.^38^ The CD spectra of AFPep1 confirmed its secondary structure by a positive peak at 199 nm and a negative peak at 208 nm (Figure 1d).^39^ The peaks at 222 and 228 nm are related to the aromatic side chains of Phenylalanine, DOPA, and Threonine.^39,40^ For the peptides AFPepC1, AFPepC2, and AFPepC3, all having the same antifreeze motif as the peptide AFPep1, both FT-IR and CD analyses displayed peaks within the same range in the spectra, corresponding to a β-sheet structure as observed for AFPep1. The secondary structure of these peptides was identified as a β-sheet structure (Figure S2a–f). The lack of prominent peaks above 220 nm in the CD spectrum of AFPepC1 can be ascribed to the absence of aromatic residues in its sequence, particularly phenylalanine. The control peptide, AFPepC4, where threonine residues were substituted with alanine, had a disordered structure according to FT-IR analysis, with a peak observed at 1651 cm^–1^ (Figure S2g).^38^ CD analysis further validated this structural characteristic, which exhibited a negative peak at 207 nm (Figure S2h).^41^ AFPC5, having the same sequence as AFPepC4 but without the amino acid lysine, exhibited a peak at 1624 cm^–1^, which can be ascribed to a β-sheet structure (Figure S2i). The peptide AFPepC6 displayed a β-turn structure, as indicated by a peak at 1666 cm^–1^ in the FT-IR spectra (Figure S2k).^41^ Additionally, the CD spectra showed a negative peak at 201 nm and a positive peak at 216 nm, further confirming the presence of a β-turn structure (Figure S2l).^42^ Notably, in the CD spectra, a decrease in temperature from 20 to −10 °C increased the intensity of peaks corresponding to an increase in secondary structure. This effect was observed at 200 and 208 nm for AFPep1.

For a higher structural characterization, 2D Nuclear magnetic resonance (NMR) spectra (TOCSY) were acquired for the AFPep1 at 13 °C in water. Peak maxima were picked on the spectra for 11 resolved doublets in each of the HN-Hα regions (Figure S3). The average ^3^*J*_HN–Hα_ coupling of AFPep1 was 8.05 Hz SD 0.46, suggesting a β-sheet secondary structure.^43^ AlphaFold2 Colab was used to calculate the structures of the peptide.^44^ The calculated structure of AFPep1 (residues 5–14) overlaid residues 108–117 of the crystal structure of *R. inquisitor* antifreeze protein^45^ (PDBid 4dt5) with a backbone RMSD of 1.82 Å (Figure 1e).

**Figure 2:**
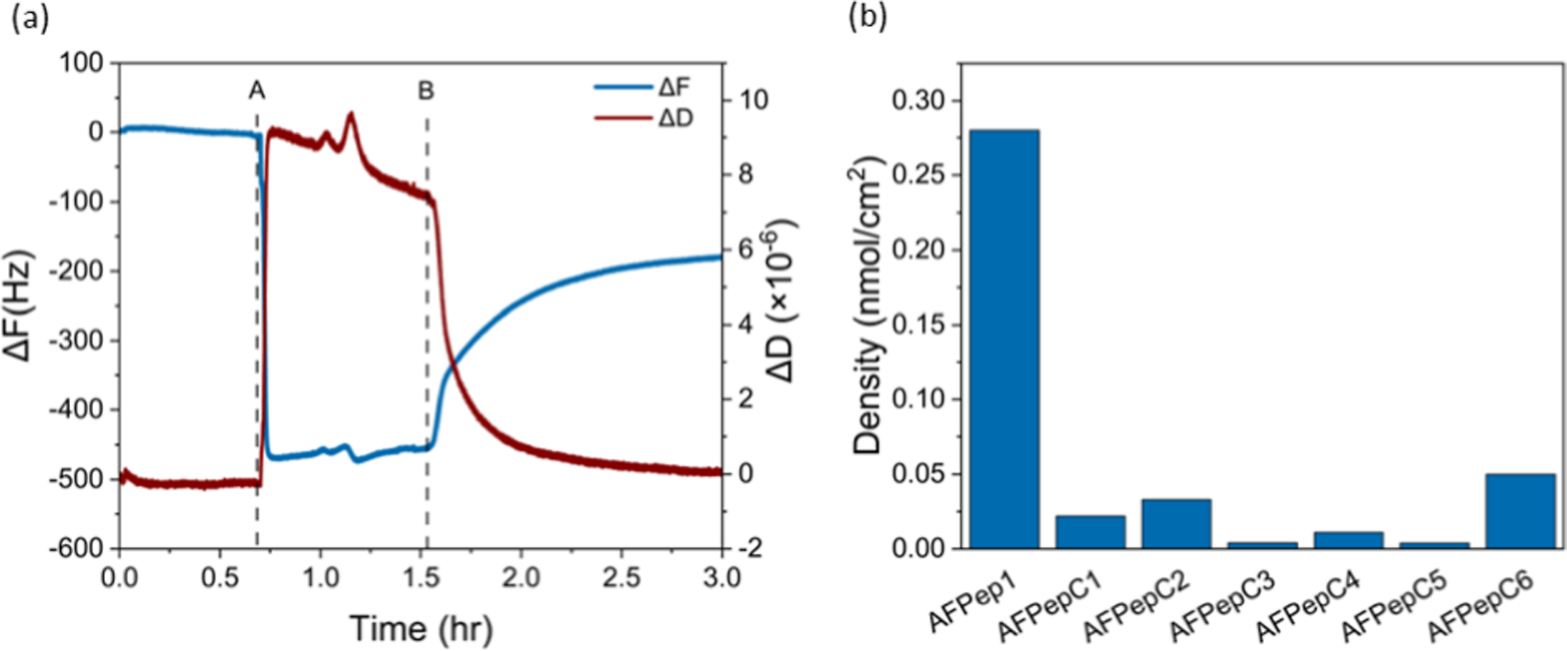
QCM analysis of the studied peptides. (a) The graph plots the change in the Si sensor frequency and dissipation while flowing a solution of AFPep1, (b) the calculated density of the peptides on the sensor.

To explore the real-time adhesion of the peptides to the surface, Quartz crystal microbalance with dissipation (QCM-D) analysis was performed. The peptide solution was circulated within a flow cell containing a silicone-coated QCM sensor. In an adhesion process, the frequency of the sensor decreases due to the growing mass of the adsorbed layer, while the dissipation increases due to the development of a film. The observed change in dissipation was approximately 0.1 × 10^–6^ for all peptides, indicating the formation of a rigid film (Figures 2a and S4). This enables the use of the Sauerbrey equation, establishing a correlation between the change in frequency and the moles of the peptide adsorbed onto the sensor.^46^

AFPep1 demonstrated the most substantial change in frequency, with a density of 0.28 nmol/cm^2^, while the change in frequency for the other peptides (AFPepC1-AFPepC6) ranged from 0.0039 to 0.050 nmol/cm^2^ (Figure 2b). This suggests strong adhesion of AFPep1 to the sensor. The improved bonding to the surface by this peptide, compared with the rest of the studied peptides can be attributed to the incorporation of the amino acid lysine next to DOPA, which is known to enhance binding to the surface.^33−35^ AFPepC2 and AFPepC5, having the same peptide sequence as AFPep1 but lacking the lysine, demonstrated a relatively small change in frequency with a density of 0.033 and 0.0039 nmol/cm^2^, respectively (Figures S4b,e, and 2b). Moreover, the peptide’s secondary structure plays a role in influencing its binding to the surface. AFPepC4, having the same peptide sequence as AFPep1 but with a threonine substitution for alanine, disrupting the anti-ice motif, shows a relatively low change in frequency, with a density of 0.011 nmol/cm^2^ (Figure S4d). The relatively low adsorption can be attributed to the disordered structure of AFPepC4, as confirmed by FT-IR and CD analysis. The impact of the diphenylalanine residues on the formation of the peptide coating was seen through the AFPepC1, having the same peptide sequence as AFPep1 but without the diphenylalanine residues while maintaining the same secondary structure. The absence of diphenylalanine residues in AFPepC1 resulted in a relatively minor change in frequency with a density of 0.022 nmol/cm^2^ (Figure S4b). The significance of DOPA is evident in AFPepC3, which lacks this amino acid. This peptide exhibited a relatively small change in frequency with a density of 0.0040 nmol/cm^2^ (Figure S4c). These findings highlight the significance of including all three elements in the peptide sequence: lysine, DOPA, and diphenylalanine, alongside the secondary structure.

**Figure 3:**
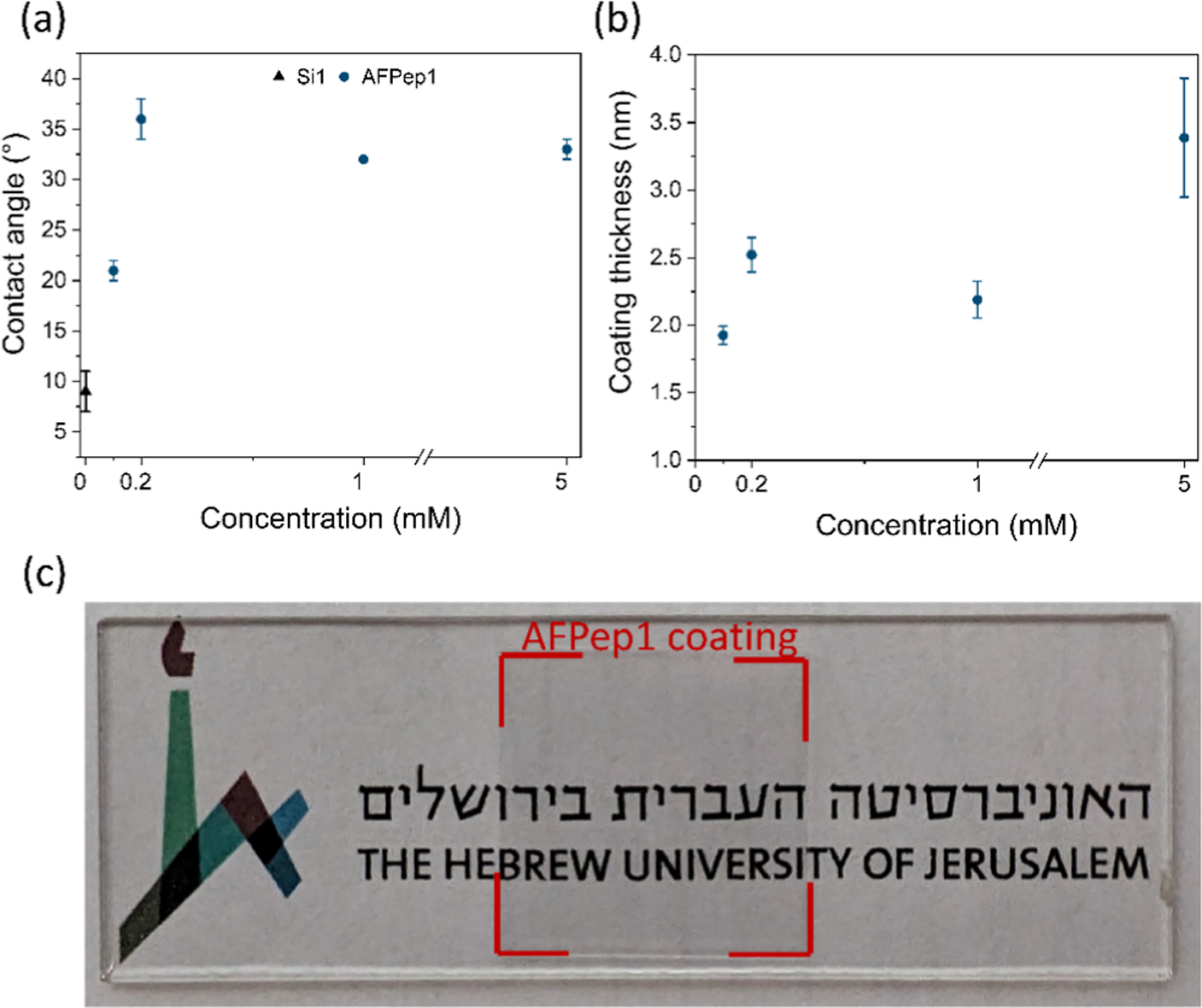
Characterization of the peptide coating. (a) Contact angle of the silicon surface and coated silicone surface with AFPep1. (b) Coating thickness of the coated silicone surface with AFPep1 at different concentrations (0.1, 0.2, 1, and 5 mM). The standard deviation (SD) was calculated based on data from three independent surfaces, each conducted in three locations. (c) Image of a 2 × 2 cm glass surface coated with AFPep1 at a concnetration of 5 mM.

To further confirm the peptide coating on the silicon surface and to evaluate the effect of peptide concentration on the coating, contact angle measurements were conducted on uncoated and peptide-coated silicone surfaces (Figure 3a). A surface coated with AFPep1 displayed an increase in the contact angle from 9 ± 2° for a clean silicon surface to 33 ± 1, 32 ± 0, 36 ± 5, and 21 ± 1° for peptide concentration of 5, 1, 0.2, and 0.1 mM, respectively. While the peptide coatings influenced the contact angle of the surface, there was no clear correlation between peptide concentration and contact angle. The control peptides (AFPepC1-AFPep6) exhibited an increase in contact angle compared to the silicon surface (Figure S5). For control peptides AFPepC3-AFPepC5, this increase exceeded that observed with AFPep1, which can be attributed to the absence of charged amino acids (e.g., lysine) or the replacement of the polar amino acid threonine with the hydrophobic amino acid alanine.

The peptide coating thickness was evaluated using an ellipsometer. The analyzed measurements were based on fitting to the Cauchy dispersion model. The thickness of the coatings for AFPep1 at various concentrations ranged from 1.9 to 2.5 nm. Remarkably, the coating of AFPep1 at a concentration of 5 mM exhibited the greatest thickness of 3.4 ± 0.4 nm (Figure 3b).

To verify the transparency of the AFPep1 coating at different concentrations, transmittance measurements were conducted. The peptide coating demonstrated exceptionally high transmittance levels, surpassing 98.7%, indicating a notably transparent surface (Figures S6 and 3C).

The stability of the coating was assessed under several environmental conditions by immersing the coated surfaces in solutions designed to simulate acidic or alkaline rain, specifically, we used either an acidic solution at pH 5 or an alkaline solution at pH 9. Additionally, the coated surfaces underwent an abrasion test against sandpaper. After these treatments, the surface’s elemental composition was analyzed using X-ray photoelectron spectroscopy (XPS). After the treatments, XPS analysis revealed no decrease in the atomic ratio of N/Si, indicating that the peptide coating remained intact (Figure S7).

**Figure 4:**
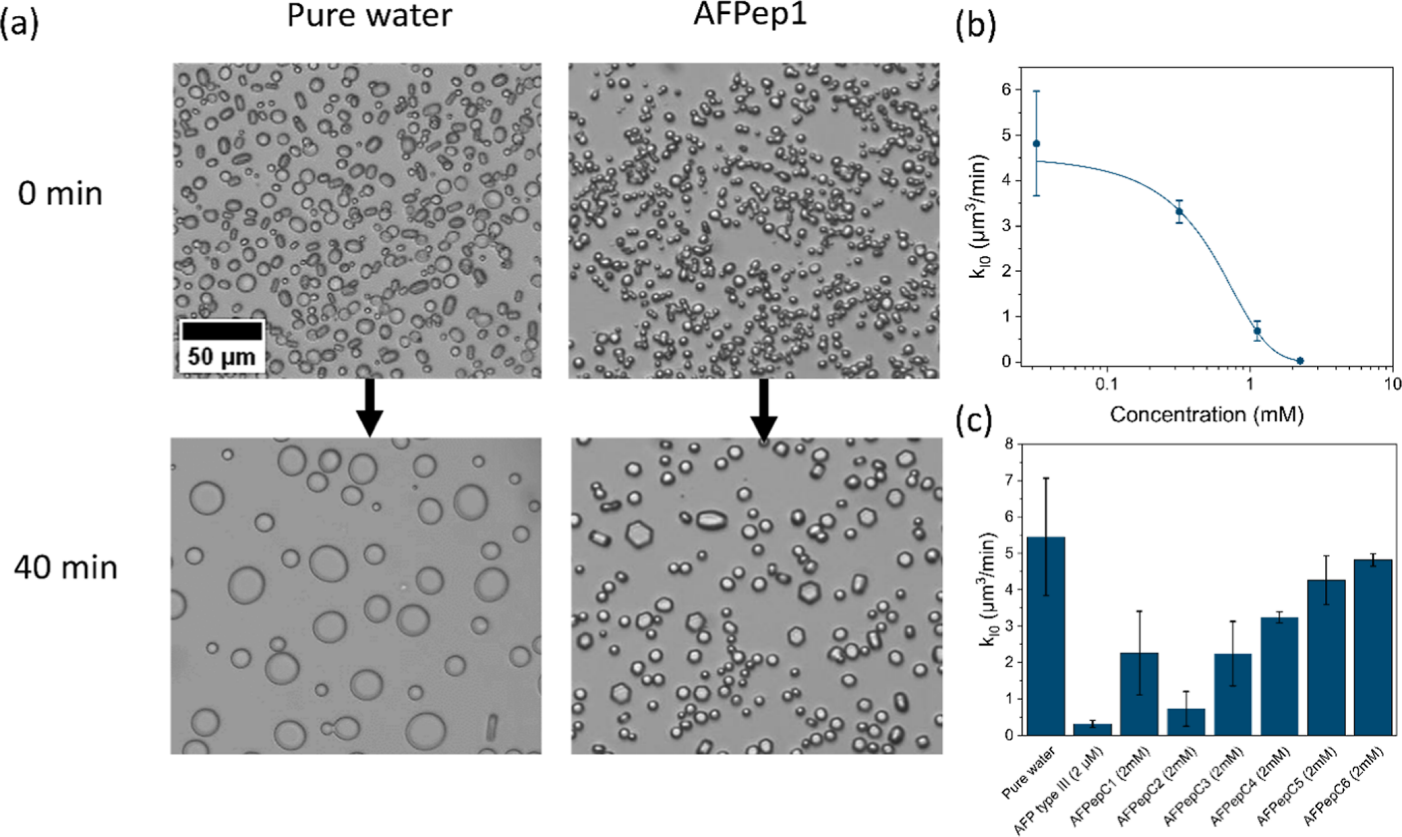
Ice recrystallization inhibition analysis. (a) Microscopic images of ice crystal recrystallization at −8 °C in 45% sucrose solutions, including pure water and AFPep1. The scale bar applies to all images. (b) Ice recrystallization rate constant at zero ice fraction for AFPep1 as a function of concentration. (c) Ice recrystallization rate constant at zero ice fraction for sucrose 45 wt %, AFP type III at 2 μM, and control peptides (AFPepC1-AFPepC6) at 2 mM. The SD was calculated from data obtained from a minimum of three independent experiments.

The rate of the ice recrystallization was evaluated by freezing the mixed sample of the peptide with sucrose solution. Afterward, the frozen sample was warmed to the annealing temperature of −8 °C for 40 min, and the crystal size was monitored over time. Figures 4a and S8 show the ice crystal growth in the presence of pure water, AFPep1, AFPepC1-AFPepC6 at 2 mM, and AFP type III at 2 μM. AFP type III represents a fully active AFP, analogous to the wild type RiAFP. The control sample of pure water yielded a final crystal radius of 9 ± 6 μm after 40 min. AFPep1 demonstrated considerable inhibition of ice crystal growth, leading to a final radius of 4 ± 2 μm. Furthermore, the presence of AFPep1 in the solution altered the shape of the ice crystals from disk-like to hexagonal plates, indicating a direct binding of this peptide to prism planes and potentially to basal planes.^47^ This high affinity is attributed to the formation of supramolecular assemblies of regularly spaced threonine amino acids, forming the “TxT” motif.^48^ The hydroxyl groups of the two threonine residues within each TxT-repeat mimic the arrangement of oxygen atoms in ice, aligning perfectly with the planes of ice and thereby reducing the temperature required for ice crystal growth.^26^

To determine the recrystallization rate of ice crystal growth, the ice volume fraction was calculated throughout the entire experiment using eq 3. Figure S9 shows the results of the ice volume fraction for the water and AFPep1. A linear growth function was used to fit the data (Figure S9), suggesting that the kinetics of the ice recrystallization process are closely described by a bulk diffusion process of water molecules from smaller to larger ice crystals, consistent with LSW theory. Then, the *k*_I_(*Q*) was assessed by analyzing the slope of the graph depicting the crystal radius over time using eq 4. The ice volume fraction, which influences the recrystallization rate, varied among the samples. To address this variation, we calculated the normalized recrystallization rate of crystal growth concerning the ice volume fraction (*k*_I0_) using eqs 5 and 6. The results of this analysis for AFPep1 at different concentrations are presented in Figure 4b. For AFPep1, the data can be fit to a sigmoid curve using eq 7, and the inflection point in the curve, representing the inhibitor concentration, can be extracted. The 50% inhibition concentration for AFPep1 is 0.5 ± 0.1 mM. Furthermore, the recrystallization end point which is the approximate concentration when *k*_I0_ equals zero at the experimental resolution was estimated from this curve. For AFPep1 it was found to be approximately 2.3 mM. Based on the classification conducted previously on various ice recrystallization inhibition (IRI) active materials, it is noted that the IRI activity of AFPep1 is considered relatively effective among common antifreeze materials (Figure S10).^49^ We note that the peptide’s self-assembly properties probably indicate that the actual concentration of the particles is lower than the 500 μM we reported. While AFPep1 is not as effective as the antifreeze glycoproteins, it has a clear IRI as an effective IRI peptide.

The results of the *k*_I0_ for the AFP type III at 2.2 μM and for the control peptides at a concentration of 2.2 mM (AFPepC1- AFPepC6) are shown in Figure 4c. The control peptides (AFPepC1- AFPepC3) having the antifreeze sequence exhibited relatively higher values of *k*_I0_ compared to AFPep1. This higher *k*_I0_ could be related to the absence of key elements: diphenylalanine, crucial for promoting self-assembly into the long fibril structures observed in AFPep1, or lysine which is known to greatly enhance solubility. This reduced solubility might result in peptide aggregation and precipitation over time. The control peptides lacking an antifreeze sequence (AFPepC4- AFPepC6) exhibited *k*_I0_ values closely resembling those of the pure water sample, as expected.

The effect of the formed peptide assemblies on the rate of ice crystal growth was analyzed using HAADF-STEM, with the resulting images shown in Figure S12. Among all the controls with the antifreeze sequence, only AFPepC2, which exhibited *k*_I0_ values close to those of AFPep1, displayed long fibril structures similar to those observed in AFPep1 (Figure S12c).

**Figure 5:**
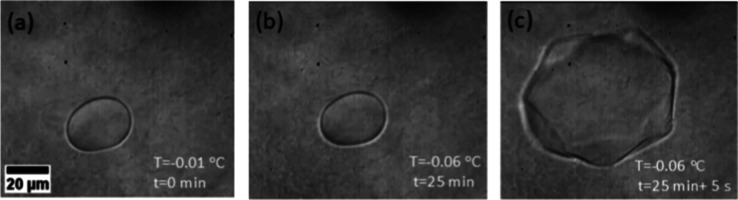
Thermal hysteresis measurement of AFPep1. (a–c) A demonstration of the ice growth during slow cooling in 10 mM AFPep1. While cooling slowly from −0.01 to −0.06 °C no growth is observed over 25 min, while sudden growth is observed over a few seconds, indicating the burst temperature. The difference between the burst temperature and the highest temperature without melting is the measure of the TH. The scale bar applies to all images. A movie of the burst of the crystal is shown in the Supporting Information, Movie S1.

The thermal hysteresis (TH) of the peptide AFPep1 and the control peptides (AFPepC1-AFPepC6) at a concentration of 2.2 Mm and 10 mM was measured using a nanoliter osmometer (Figure S13). AFPep1 displayed a TH of 0.040 ± 0.002 °C at 2.2 mM, which increased to 0.050 ± 0.02 °C at 10 mM (Figure 5).

Notably, the control peptide AFPepC1, which contains the antifreeze sequence but lacks the diphenylalanine, showed a negligible TH of 0.002 ± 0.001 °C at 2.2 mM and a significantly higher TH of 0.20 ± 0.01 °C at 10 mM. The substantial increase in TH for AFPepC1 at higher concentration can be attributed to concentration-dependent aggregation, as confirmed by cryo-TEM analysis, which revealed the formation of fiber aggregates at 10 mM and the absence of such aggregates at concentration of 2.2 mM (Figure S14c,d). These aggregate structures are presumed to be stable^50^ and likely enhance antifreeze activity by increasing the density and spatial organization of exposed antifreeze motifs, thereby facilitating more effective ice-binding. In contrast, at 2.2 mM, the absence of such aggregates indicates that the peptide remains predominantly monomeric, resulting in negligible TH activity.

Additionally, peptides AFPepC2 and AFPepC3, which contain diphenylalanine residues but lack lysine, exhibited lower TH values at concentrations of 2.2 and 10 mM. This reduction in TH is likely due to decreased solubility associated with the absence of lysine, resulting in peptide aggregation and eventual precipitation over time. The control peptides lacking an antifreeze sequence (AFPepC3- AFPepC6) exhibited negligible TH, further emphasizing the importance of specific sequence elements in antifreeze functionality.

**Figure 6:**
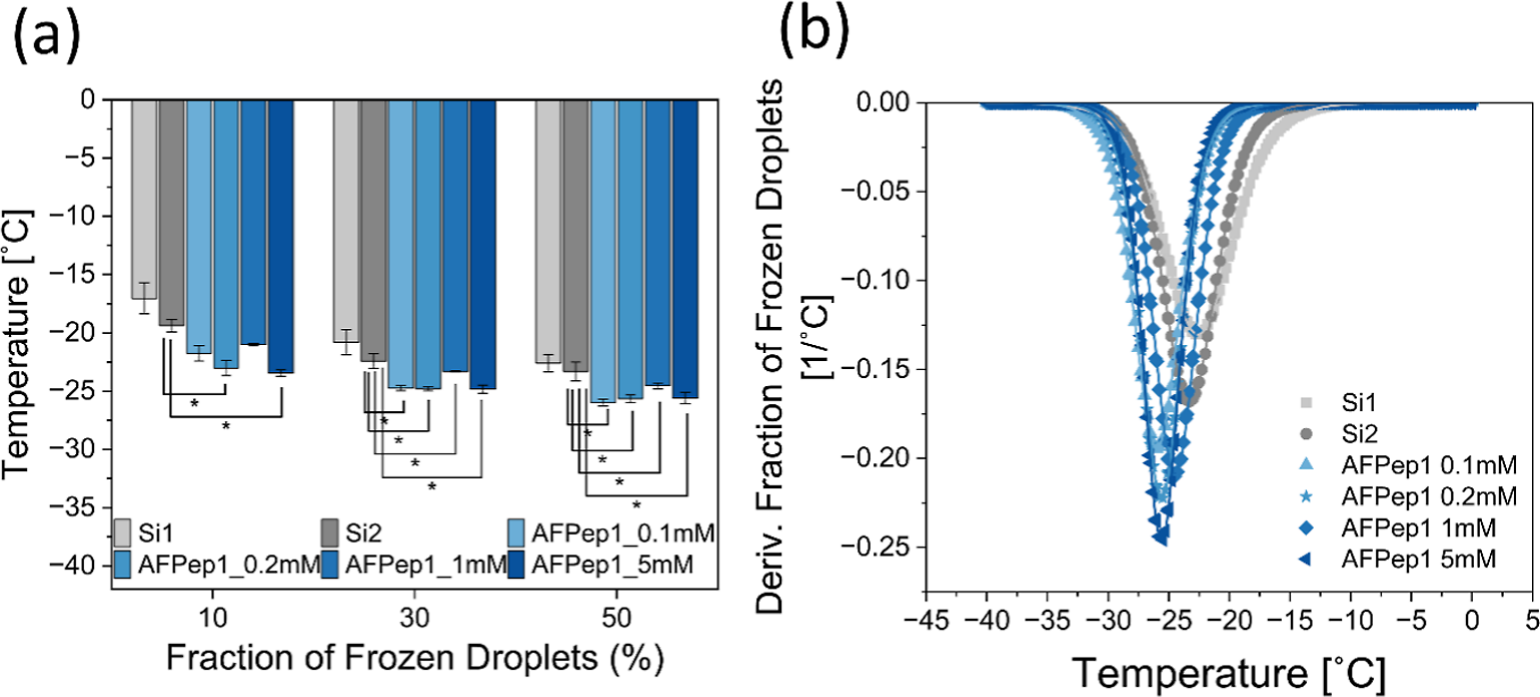
Antifreeze activity of peptide-coated surfaces. (a) Temperatures correspond to selected fractions of frozen droplets (10%, 30%, and 50%) for AFPep1. The standard error was calculated using data from at least three independent experiments. An ANOVA test followed by a Tukey–Kramer post hoc analysis was conducted to identify statistically significant differences between the Si2 surface and peptide-coated surfaces. Results were considered statistically significant at *p* < 0.05 and are indicated with an asterisk (*). (b) Derivative of the fraction of frozen droplets for the control surfaces and AFPep1.

In the droplet freezing assay, droplets of 1 μL water were pipetted onto clean silicon wafers (Si1 and Si2), as well as silicon wafers coated with AFPep1 at a concentration of 0.1, 0.2, 1, and 5 mM. The wafers were then frozen at a controlled rate until they reached −40 °C, with the entire process being filmed continuously. The changes in the frozen droplet fraction per temperature were then observed for each coating (Figure S15). The observed results showed different freezing behavior: Si1 froze at the highest temperature, followed by Si2, while the peptide coatings of AFPep1 froze at a lower temperature. The first factor to inspect the antifreeze activity is the temperature at which 10% (*T*_10%_), 30% (*T*_30%_), and 50% (*T*_50%_) of the droplets froze (Figure 6a). For AFPep1 coatings, *T*_10%_ ranged from −21.0 ± 0.1 to −23.5 ± 0.3 °C compared to Si1 with −17 ± 1 °C and Si2 with −19.4 ± 0.5 °C. The AFPep1 coatings at a concentration of 0.2 and 5 mM were statistically significant compared to Si2. For AFPep1, the *T*_30%_ of the coatings at 0.1, 0.2, and 5 mM were around −24.8 °C, while the 1 mM coating exhibited a higher temperature at −23.3 ± 0.1 °C. These results were compared to the clean wafers, which showed freezing temperatures of −21 ± 1 °C for Si1 and −22.4 ± 0.6 °C for Si2, with statistically significant differences. The *T*_50%_ values were −22.6 ± 0.8 °C for Si1 and −23.3 ± 0.8 °C for Si2. In contrast, the AFPep1 coatings had *T*_50%_ values ranging from −24.5 ± 0.3 to −26.0 ± 0.2 °C. The AFPep1 exhibited the lowest temperatures at the 0.1 mM coating concentration, with all coatings showing statistically significant differences compared to Si2.

While it was expected that an increase in surface roughness would lower the antifreeze activity,^51^ the results exhibited a different trend. The AFPep1 coatings, which had the highest roughness values (Table S1), also showed the highest nucleation depression activity. It can be concluded that with no clear trend regarding the effect of contact angle or surface roughness, the nucleation depression activity is primarily due to the peptide coating itself.

Each surface’s droplet freezing rate was calculated to further evaluate the antifreeze activity. A fitted curve was generated for each coating. Then, the curve’s derivative as a function of temperature was calculated (Figure 6b). It can be seen that AFPep1 peptide coatings delayed drop freezing by approximately 5 °C compared to the control surfaces (Si1 and Si2). Moreover, the rate of drop freezing as a function of temperature [factor *f* in eqs 8 and (9)] ranged from −0.20 °C^1–^ to −0.25 °C^1–^ for AFPep1. This can be explained by the self-assembly of the peptide into structures that interact effectively with ice crystals, attributed to the unique TXT motif sequence present in AFPep1. This interaction is further supported by the IRI and TH activity associated with AFPep1. The IRI activity slows down ice crystal growth, which contributes to the enhanced resistance of AFPep1 to lower temperatures.

## Conclusions

In this study, we designed a peptide incorporating antifreeze motifs derived from antifreeze proteins from the beetle *R. inquisitor.* The peptide, AFPep1, can spontaneously self-assemble in water into fibrous or spherical shapes on surfaces and fibrous structures in solution. The presence of the amino acids DOPA, Lysine, and diphenylamine, contributed to the formation of an environmentally friendly and durable coating. The antifreeze activity of AFPep1 is attributed to the TxT-repeat, which mimics the arrangement of oxygen atoms in ice, allowing the protein to align perfectly with ice planes and inhibit ice crystal growth, thereby lowering the freezing point. The reduced recrystallization inhibition and TH of the peptide lacking phenylalanine residues at 2.2 mM suggest that the peptide’s activity depends on its ability to assemble and bind effectively to ice. At higher concentrations (10 mM), DOPA alone induces aggregation, resulting in significant TH activity. However, at lower concentrations, the combination of phenylalanine with DOPA is necessary for effective surface coating. Thus, while phenylalanine is not required at high concentrations for TH activity, it plays a crucial role in surface coating at lower concentrations, providing the desired benefits of both coating and ice activity. One limitation of this work is that we did not conduct a direct comparison of the designed peptide with the native antifreeze protein from *R. inquisitor*. Nevertheless, the designed peptide holds great potential in various fields such as cryopreservation, anti-icing coatings, and the development of frost-resistant materials for industrial and biomedical applications.

## Materials and Methods

### Materials

Fmoc-DOPA(acetonide)–OH, Fmoc-Pro-OH, Fmoc-Thr(*t*Bu)–OH, Fmoc-Gln(Trt)-Oh, Fmoc-Ile-OH, and NH_2_-DOPA-Phe-Phe-OMe were purchased from GL Biochem (Shanghai, China). Fmoc-Gly-OH, Fmoc-*l*-Lys(Boc)–OH, Fmoc-*l*-Phe–OH, rink amide AM resin, and ethyl cyanohydroxyiminoacetate (oxyma) were obtained from Matrix Innovation (Quebec, Canada). *N*-Fmoc-*l*-Ala was purchased from Thermo Fisher Scientific. *N*,*N*′-Diisopropylcarbodiimide (DIC), sodium dodecyl sulfate (SDS), sucrose, deuterium oxide (D_2_O), and alpha-cyano-4-hydroxycinnamic acid (α-Cyano) were purchased from Sigma-Aldrich (St. Louis, Mo, USA). Triisopropylsilane (TIPS) was purchased from TCI (Kita-Ku, Tokyo, Japan). Dimethylformamide (DMF), dichloromethane (DCM), diethyl ether, trifluoroacetic acid (TFA), piperidine, acetonitrile, and ultrapure water LC/MS grade were purchased from Bio-Lab Itd (Jerusalem, Israel). Triple distilled water (TDW) was obtained by filtering distilled water through a Milli-Q water system (Millipore). Immersion Oil Type B was purchased from Cargille Laboratories (Cedar Grove, USA). AFP Type III, derived from the ocean pout (Macrozoarces americanus) fish, was produced through expression in *E. coli*.

### Substrates

Silicon wafers with a diameter of 10 cm were diced into 0.8 × 0.8 cm^2^ pieces before use (7100 2 in. Pro-Vectus, ADT).

### Synthesis of the Peptides

The peptides were synthesized by using a Liberty blue microwave-assisted peptide synthesizer (CEM) utilizing standard Fmoc-SPPS chemistry. Rink amide resin was used and swelled in DMF for 30 min at ambient temperature before the synthesis. Fmoc deprotection was done with 5 mL of 20% piperidine solution in DMF for 1 min at 90 °C. A double coupling of amino acids (0.2 mM) with DIC (1.0 M) and Oxyma (1.0 M) reagents for 8 min at 90 °C. Washes between deprotection and coupling steps involved DMF with a 5 s drain time, a total of 13 mL. Following the completion of the synthesis, the peptide resin underwent washing with DMF (three repetitions) and DCM (three repetitions), followed by drying under vacuum for 3 min. The peptide resin was suspended in a cleavage solution comprising a mixture of TFA/TDW/TIPS (95:2.5:2.5) totaling 20 mL and mixed for 3 h at ambient temperature. Then, the TFA was evaporated by bubbling nitrogen, and the crude peptide product was precipitated with diethyl ether. After centrifugation (10 min, 4000 rpm), diethyl ether was decanted, and the peptide was dissolved in TDW and subjected to lyophilization.

### High-Performance Liquid Chromatography

The peptide purity was evaluated through analytical reversed-phase (RP) high-performance liquid chromatography (HPLC) using a Waters Alliance system with UV detection at 220 and 280 nm. The peptide was injected into the XSelect C18 column (3.5 μm, 130 Å, 4.6 mm × 150 mm). The peptides were purified through preparative RP-HPLC by using an Ultimate 3000 HPLC system (Thermo-Fisher Scientific), equipped with a C18 LC column (10 μm, 110 Å, 250 × 21.2 mm) and with UV detection at 220 and 280 nm. In both systems, peptide elution occurred through a linear gradient from 5% to 70% acetonitrile (with 0.1% TFA) in water (with 0.1% TFA). The flow rate was set at 1 mL/min and 30 °C for analytical RP-HPLC and 20 mL/min at room temperature for preparative RP-HPLC (Figures S16–S21).

### Mass Spectrometry

The fractions obtained during the RP-HPLC were analyzed using a mass spectrometer (Bruker MALDI-TOF MS Autoflex, Germany) in positive ion mode. The mass range analyzed was from 400 to 2000 *m*/*z*. For the MALDI matrix, a 25 mM solution of α-Cyano was prepared in a solution consisting of 50% water/acetonitrile and 0.1% TFA. The peptide samples were mixed with the α-Cyano solution at a 1:1 volume ratio, placed on the MALDI plate, and then dried for analysis (Figures S16–S21).

### Preparation of the Peptide Coating

Before peptide-coating, silicon surfaces (0.8 cm × 0.8 cm) underwent a series of cleaning treatments. Initially, they were exposed to a UVO chamber (Jelight Company, USA) for 10 min, followed by soaking in 2% SDS in TDW for 30 min. Subsequently, they were washed with TDW, dried using N_2_ gas, and subjected to a 30 s treatment with Oxygen/Plasma (Atto, Diener Electronic). The treated silicon surfaces (Si1) were then immersed in 200 μL of peptide solution (at concentrations of 5, 1, 0.2, and 0.1 mM in LC/MS water) in 48 well plates and sealed overnight at room temperature. Following the peptide-coating process, the excess of the unattached peptide on the silicon surfaces was removed by pipetting 2 mL of LC/MS water, followed by drying under N_2_ gas. The coated surfaces were kept in a desiccator until the measurements were conducted.

### Atomic Force Microscope

The surface topography and roughness of the silicon surfaces were analyzed using atomic force microscopy (AFM). The measurements were conducted on a Dimension Icon-XR SPM system (Bruker, USA) operating in tapping mode with an RTESP probe (*F* = 300 kHz, *k* = 42 N/m).

### Cryo-Transmission Electron Microscopy

The peptide morphology was analyzed using cryo-TEM. Initially, a 300-mesh copper grid coated with a holey carbon film (Lacey substrate, Ted Pella, Ltd.) underwent pretreatment via glow discharge (30 s in the plasma). Subsequently, a drop of peptide solution at a concentration of 5 mM in TDW was applied to the treated TEM grid. The specimens were rapidly vitrified by plunging into liquid ethane precooled with liquid nitrogen, maintaining controlled temperature and 100% relative humidity, utilizing a Vitrobot Mark IV. These vitrified samples were then transferred to a cryo specimen holder (Gatan model 626; Gatan Inc.) and imaged at −179 °C using a Tecnai 12 G^2^ Twin TEM (FEI), operated at an acceleration voltage of 120 kV in low-dose mode. Images were recorded using a 4 K × 4 K FEI Eagle CCD camera.

### Circular Dichroism

Circular dichroism (CD) spectra were obtained using a J-1100 spectropolarimeter (JASCO, Tokyo, Japan) equipped with a 0.1 cm path length quartz cuvette for far-UV CD spectroscopy. Measurements were conducted at temperatures 20, 10, 0, and −10 °C within the spectral range of 190–260 nm, with a step width of 0.1 nm. The samples were dissolved in TDW to achieve a final peptide concentration of 0.3 mM and were subsequently filtered using a 0.22 μm filter. For each sample, five spectra were gathered, averaged, and background-subtracted utilizing TDW as the baseline.

### Fourier-Transform Infrared Spectroscopy

A 20 μL droplet of peptide solution, with a concentration of 5 mM in D_2_O, was deposited onto CaF_2_ plates and subsequently subjected to vacuum drying. The FT-IR spectra were acquired using a Nicolet 6700 FT-IR spectrometer equipped with a deuterated triglycine sulfate (DTGS) detector (Thermo Fisher Scientific, MA, USA). Spectra were collected over a range of 400–4000 cm^–1^ with a resolution of 4 cm^–1^. To ensure precision in data, the spectra were averaged after 2000 scans.

### Nuclear Magnetic Resonance

Nuclear magnetic resonance (NMR) experiments were performed on a Bruker AVII 500 MHz spectrometer operating at the proton frequency of 500.13 MHz, using a 5 mm selective probe equipped with a self-shielded *xyz*-gradient coil at 13 °C. The transmitter frequency was set on the water signal. Two-dimensional spectra (TOCSY) were used to achieve the resolution of the HN-Hα peaks. Spectra were processed with TopSpin (Bruker Analytische Messtechnik GmbH) and NMRFAM SPARKY software was used to identify peak maxima.^52^ GraphPad online was used to perform an unpaired *t*-test to determine statistical significance.

### Peptide Structure Prediction

AlphaFold-Multimer-v2.0 Colab was used to calculate the structures of the peptide.^44,53^

### Quartz Crystal Microbalance with Dissipation

The adhesion of peptides to the silicon surface was examined using QCM-D (Q-sense, Biolin Scientific). Measurements were conducted in a flow module E1 system featuring SiO_2_ sensors with a fundamental resonant frequency of 5 MHz (Qsense). Before each experiment, the silicon sensors were cleaned following the supplier’s instructions. The experiments were carried out under flow-through conditions utilizing a digital peristaltic pump (IsmaTec Peristaltic Pump, IDEX). Initially, TDW was circulated into the sensor crystal chamber at a rate of 0.1 mL/min until a stable frequency and dissipation were achieved. Subsequently, the peptide solution, with a concentration of 0.6 mM dissolved in TDW, was injected into the sensor crystal chamber at the same rate for approximately 1 h. Following peptide injection, the sensor was rinsed with TDW to remove nonadherent peptides. The adsorbed mass (Δ*m*) was then calculated using the Sauerbrey equation, utilizing the seventh overtones
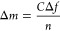
1where Δ*f* is frequency change during adsorption, *C* is a sensitivity constant characteristic of quartz crystal and is equal to 17.7 ng·cm^–2^·Hz^1–^, and n is the overtone number.

### Contact Angle Measurements

The water contact angle was measured using a Theta Lite Optical Tensiometer (Attension Theta, Finland). The volume of each drop was 1.0 μL. The measurements were averaged at three different locations on at least three independent surfaces.

### Transmittance Measurements

The transmittance of peptide-coated glass surfaces at various concentrations (5, 1, 0.2, and 0.1 mM) was measured using a spherical haze meter (Diffusion Systems Ltd., England). The uncoated glass was used as the reference. For each concentration, three surfaces were prepared, and their transmittance was measured and averaged.

### Ellipsometer

The thickness of peptide-coated silicon surfaces at various concentrations (5, 1, 0.2, and 0.1 mM) was evaluated by using an ellipsometer (J.A. Woollam, Lincoln, Nebraska, USA). The analysis was conducted at wavelengths ranging from 380 to 900 nm and at a 70° angle of incidence. The Cauchy dispersion model was employed to fit the thickness of the layers and refractive indices.^54^ Initially, the coefficients of the Cauchy equation were fixed for organic layers (*A*_n_ = 1.45, *B*_n_ = 0.01, and *C*_n_ = 0), with allowance for an angle offset. Subsequently, these parameters were adjusted to achieve more precise values.

### Stability of the Peptide Coating

The stability of the peptide coating under environmental conditions was evaluated by immersing the silicon peptide-coated surface in HCl at pH 5 or NaOH at pH 9 for 20 min. Additionally, the coated surface underwent an abrasion test. In this test, the sample was pressed with a weight of 25 g against sandpaper (2000 grit size). It was then moved in a straight line for a distance of 10 cm. After each treatment, the surfaces were washed by pipetting 2 mL of LC/MS water, then drying under N_2_ gas. Each sample underwent three repetitions.

### X-ray Photoelectron Spectroscopy

X-ray photoelectron spectroscopy (XPS) measurements were carried out using a Kratos AXIS Supra spectrometer (Kratos Analytical Ltd., Manchester, U.K.) with Al Kα monochromatic radiation X-ray source (1486.6 eV). The XPS spectra were acquired with a takeoff angle of 90°, normal to the analyzer, under a vacuum condition of 2 × 10^–9^ Torr. The survey spectra were assessed using a pass energy of 160 eV and a step size of 1 eV. High-resolution XPS spectra were measured using a pass energy of 20 and a step size of 0.1 eV. The binding energies were calibrated using C 1s peak energy as 285.0 eV. High-resolution XPS spectra were obtained for the Si 2p, O 1s, C 1s, and N 1s peaks using a pass energy of 20 eV and a step size of 0.1 eV. The data were collected and analyzed with the ESCApe processing software (Kratos Analytical Ltd.) and CasaXPS (Casa Software Ltd.).

### Ice Recrystallization Inhibition

The sample was dissolved in LC/MS grade water and mixed with a 60 wt % sucrose solution to create a solution containing 45% sucrose. A 2 μL droplet of this solution was sandwiched between two glass coverslips and sealed with immersion oil to prevent evaporation. Then, it was positioned on a silver block within the Linkem cell (Linkam MDBCS 196 temperature-controlled cold stage, Linkam Scientific Instruments Ltd., UK). Subsequently, the temperature was decreased from room temperature to −40 °C at a rate of 10 °C/min, followed by a gradual warming to −8 °C at a rate of 5 °C/min. The sample was maintained at −8 °C for 40 min, during which images were captured every minute using a microscope (Olympus BX41) equipped with a QImaging EXi Aqua bio-Imaging microscopy camera. Each sample underwent at least three repetitions.

The average crystal area in each image (A) was analyzed by using ImageJ software. The average radius of the crystal (*r*) was calculated according to the following equation
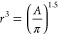
2

The ice volume fraction (*Q*) was calculated according to the following equation
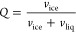
3where *v*_ice_ is the total ice volume of the crystals and *v*_liq_ is the total liquid volume.

The observed rate constant of recrystallization, *k*_I_(*Q*) was determined at a stable ice volume fraction (after 25–30 min) by calculating the slope using Lifshitz, Slyozov, and Wagner (LSW) equation
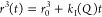
4where *r*_0_ is the initial mean crystal radius at time *t* = 0 and *r*(*t*) is the ice crystal radius at time *t*.

By using the eqs 4 and 5, the rate constant of recrystallization scaled to *Q* = 0 (*k*_I0_) was calculated.

*k*_d0_ is the apparent rate constant *k*_d_(Q) at *Q* = 0, ρ is a scaling factor that considers a variable dependence on *Q* at different temperatures. For annealing at −8 °C, *p* = 1.318 and *k*_d0_ = 0.65. α = 1 is the ratio of the mean crystal radius *r* and the critical radius.^47^
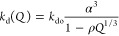
5
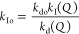
6

For the AFPep1, where the recrystallization rate was analyzed at different concentrations of these peptides, the sigmoid fit was used by the following equation
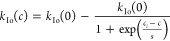
7where *k*_I0_(0) is the rate constant of 45% sucrose solution, scaled to *Q* = 0, *c*_*i*_ is the concentration in the inflection point of the curve, *C* is the peptide concentration, and *s* is the slope of the curve in the inflection region.

### High-Angle Annular Dark-Field Scanning Transmission Electron Microscopy

The copper grids (200 mesh carbon coated with Formvar, Ted Pella, Inc.) were treated with O_2_ plasma for 30 s. Following this treatment, a 1.5 μL sample of peptide solution at a concentration of 0.2 mM in TDW was deposited onto the grids and allowed to dry under ambient conditions. Subsequently, the grids were examined using an analytical high-resolution SEM Apreo 2S (Thermo Fisher Scientific) equipped with a high-angle annular dark-field scanning transmission electron microscopy (HAADF-STEM) detector. The analysis was conducted at an operating voltage of 25 kV, a current of 0.2 nA, and a working distance of 10 mm.

### Thermal Hysteresis Evaluation

Thermal hysteresis was evaluated using a computer-controlled nanoliter osmometer constructed in our lab, as described previously.^55^ The setup consists of a Peltier-based cooling block that cools a metal disc containing 500 μm holes filled with immersion oil type B. The block includes Peltier coolers, and a thermistor is attached to the metal plate for precise temperature control, which is managed by a driver controlled by LabVIEW software. The block is mounted on a microscope, and the experiments are recorded with a video CMOS camera (DMK 23UV024, The Imaging Source). A solution containing the peptides was injected into the oil using a stretched glass capillary to form approximately 200 μm droplets. The sample was cooled until the solution nucleated, typically at temperatures lower than −25 °C, and subsequently warmed until a single approximately 50 μm ice crystal remained. The melting temperature of the crystal was recorded. The crystal was maintained a few millikelvins below its melting point for 10 min. Following this, the temperature was decreased by 0.001 °C every 20 s, corresponding to a cooling rate of 0.003 °C/min, until sudden crystal growth was observed. The difference between the growth and melting temperatures represents the thermal hysteresis gap, measured at least three times for each sample.

### Antifreeze Activity on Surfaces

Antifreeze activity on surfaces was assessed by quantifying ice nucleation by recording droplet freezing using a Grant-Asymptote instrument (Grant Technologies, EF600 M 106 cold stage, UK).^56^ Before testing, the silicon wafers were coated with AFPep1, as described in the methods section. Two types of clean silicon wafers were used as controls. The first type, Si1, was prepared following the method described in the peptide coating preparation section. The second type, Si2, involved silicon wafers that were sonicated in ethanol for 15 min, washed with water, and then immersed in LC/MS grade water overnight.

In each run, 40 silicon wafers (0.8 cm × 0.8 cm) were positioned atop a metal plate. Then, 1 μL droplets of LC/MS grade water were pipetted onto each wafer, and the plate was covered with a plastic lid. The system was isolated with a plastic box sealed with plastic foam, and recordings were captured through a hole at the top using a Microsoft webcam. The droplets, initially added at 15 °C, underwent a cooling gradient of 2 °C/min until reaching 0 °C, followed by a cooling gradient of 1 °C/min until −40 °C. The temperature changes of the cold plate were monitored using the accompanying Grant Asymptote User Software Suite (Version 3.0). The process was filmed throughout, and during analysis, the number of frozen droplets was recorded. The results of each coating were then fitted with a negative sigmoid curve based on the following equation
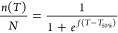
8where *n*(*T*) is the number of frozen droplets at a specific temperature, *N* is the total number of droplets (40 in each run), *f* is the droplet freezing factor, *T* is the temperature and *T*_50%_ is the temperature in which 50% of droplets were frozen.

The rate of freezing as a function of temperature was determined by calculating the derivative of eq 8 and utilizing the values of *T*_50%_ and *f*
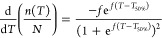
9

## Supporting Information Available

AFM images of AFPep1 at different concentrations, FT-IR and CD analysis of the peptides AFPepC1-AFPepC6, NMR TOCSY spectra of AFPep1, QCM-D measurements of AFPepC1-AFPepC6, water contact angle values for the peptides AFPepC1-AFPep C6, Transmittance of glass surfaces coated with either AFPep1 at different concentrations, XPS analysis of peptide-coated surfaces before and after treatments, microscopic images of ice crystals recrystallization at −8 °C in 45% sucrose solutions, including control peptides (AFPepC1-AFPepC6) at 2 mM and AFP type III at 2 μM, representative results for a cubic mean radius of ice crystals and ice volume fraction during IRI experiment for water and AFPep1, ice recrystallization inhibition analysis effectivity, HAADF-STEM images for the peptides, thermal hysteresis of peptides AFPep1 and AFPepC1-AFPepC6 at concentrations of 2.2 mM and 10 Mm, Cryo-TEM images of AFPep1 and AFPepC1 at different concentrations, fraction of frozen droplets at decreasing temperature for AFPep1, surface roughness of bare silicon surfaces (Si1 and Si2) and peptides AFPep1 at different concentrations, Analytical HPLC chromatograms of the synthesized peptides, mass spectrometry analysis of the synthesized peptides (WORD) (PDF)
A movie showing the burst of the crystal in solution containing AFPep1 at a concentration of 10 mM (Movie S1) (AVI)

## Supplementary Material

am4c22816_si_001.pdf

am4c22816_si_002.avi
