# Supplementary material for: Spontaneous Formation of a Sustainable Antifreeze Coating by Peptide Self-Assembly
Source: ACS Appl Mater Interfaces. 2025 Mar 2;17(10):16256–67. doi: 10.1021/acsami.4c22816 (PMC11912201; doi:10.1021/acsami.4c22816)
Supplement: Supplementary file 1 — am4c22816_si_001.pdf [file am4c22816_si_001.pdf]

Supporting information

Spontaneous Formation of a Sustainable Antifreeze Coating by Peptide Self-Assembly

Michaela Kaganovich[a,b], Eilam Gibeon[a,b], Anna Shilling Bakalinsky[e], Deborah E Shalev[c,d], Ido Braslavsky[e*] and Meital Reches[a,b*]

[a]Institute of Chemistry, The Hebrew University of Jerusalem, Jerusalem, 9190401, Israel

[b]The Center for Nanoscience and Nanotechnology, The Hebrew University of Jerusalem, Jerusalem, 9190401, Israel.

E-mail: meital.reches@mail.huji.ac.il

[c]Wolfson Centre for Applied Structural Biology, The Hebrew University of Jerusalem, Jerusalem, 9190500, Israel

[d]Department of Pharmaceutical Engineering, Azrieli College of Engineering, Jerusalem, 9103501, Israel

[e]The Robert H. Smith Faculty of Agriculture, Food and Environment, Institute of Biochemistry, Food Science, and Nutrition, The Hebrew University of Jerusalem, Rehovot, 7610001, Israel

E-mail: ido.braslavsky@mail.huji.ac.il

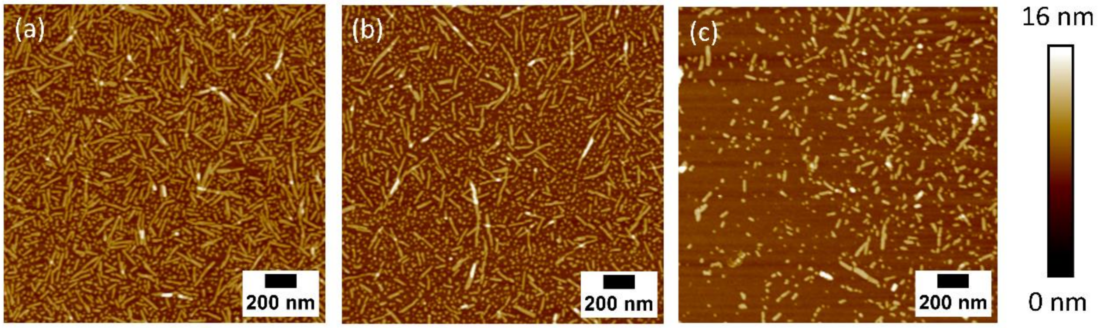

Figure S1. Representative AFM images of AFPep1 at a concentration of (a) 1 mM, (b) 0.2 mM, and (c) 0.1 mM.

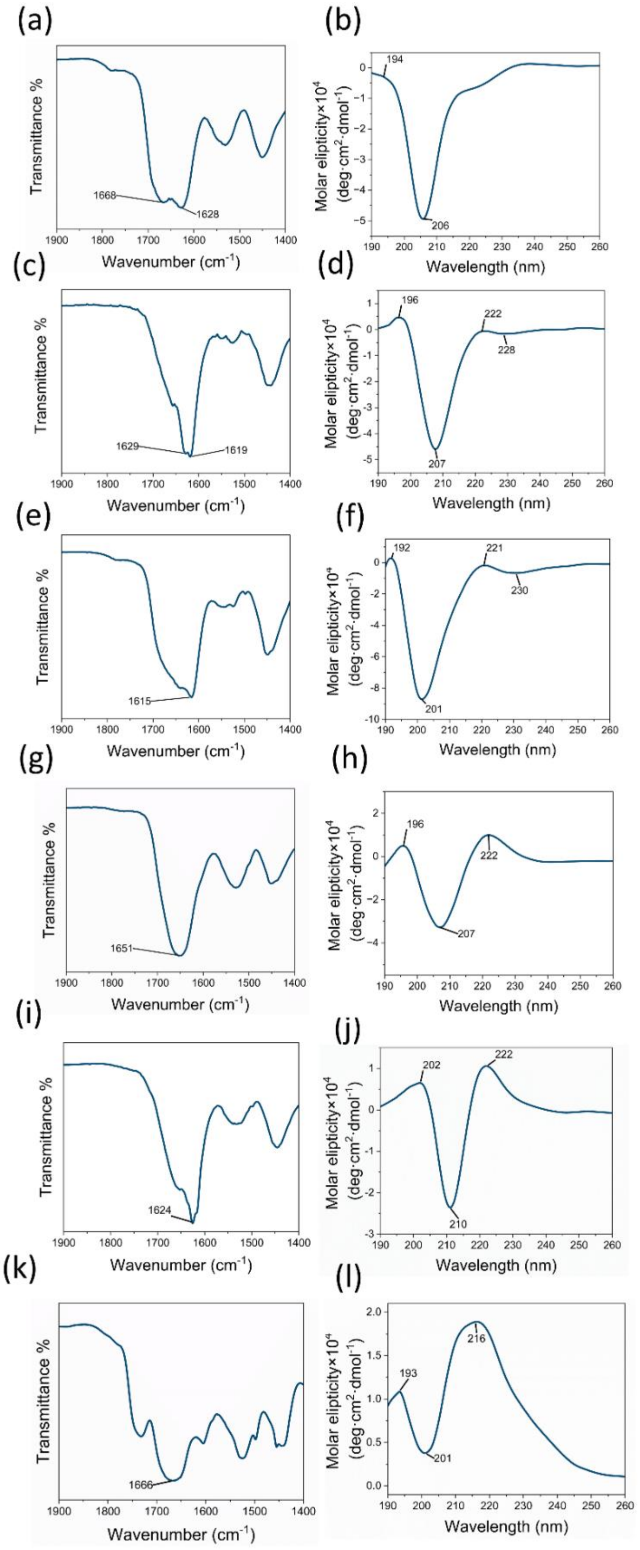

Figure S2. FT-IR and CD analysis of (a and b) AFPepC1, (c and d) AFPepC2, (e and f) AFPepC3, (g and h) AFPepC4, (i and j) AFPepC5, and (k and l) AFPepC6.

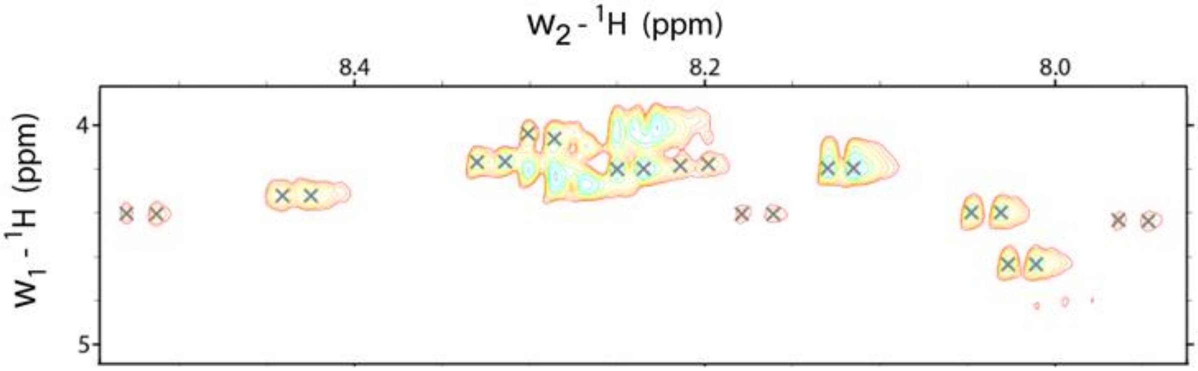

Figure S3. NMR TOCSY spectra of AFPep1 showing HN-Hα region and the unique picked peaks for $^3J_{HN\text{-}H\alpha}$ coupling determination.

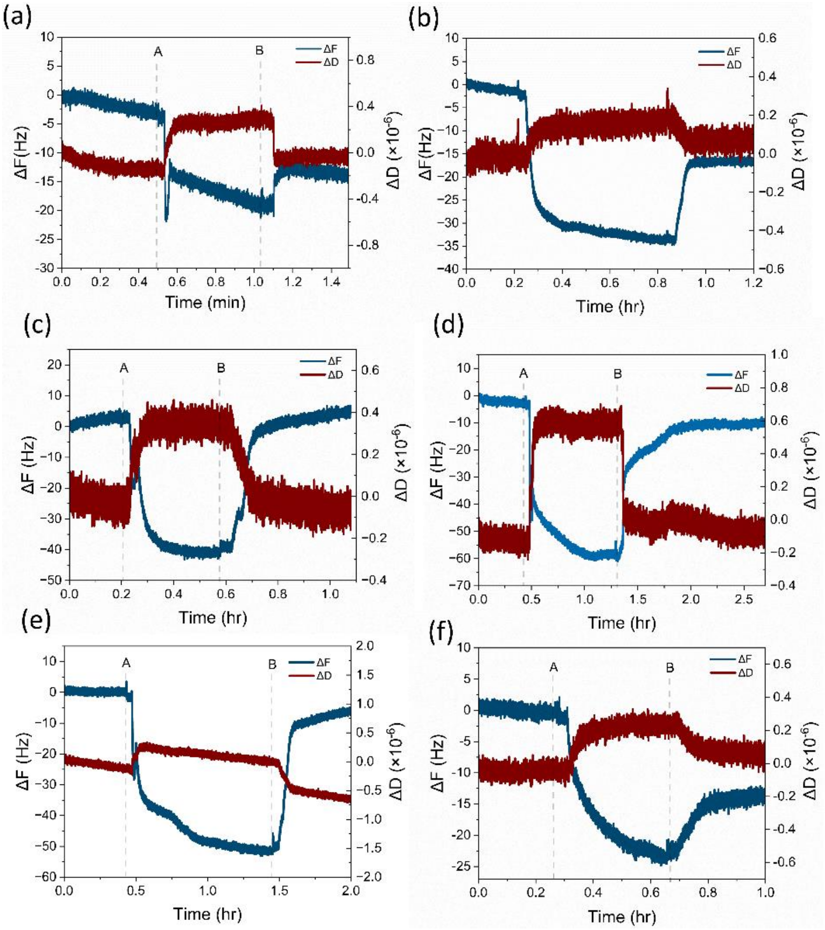

Figure S4. QCM-D measurements of (a) AFPepC1, (b) AFPepC2, (c) AFPepC3, (d) AFPepC4, (e) AFPepC5, and (f) AFPepC6.

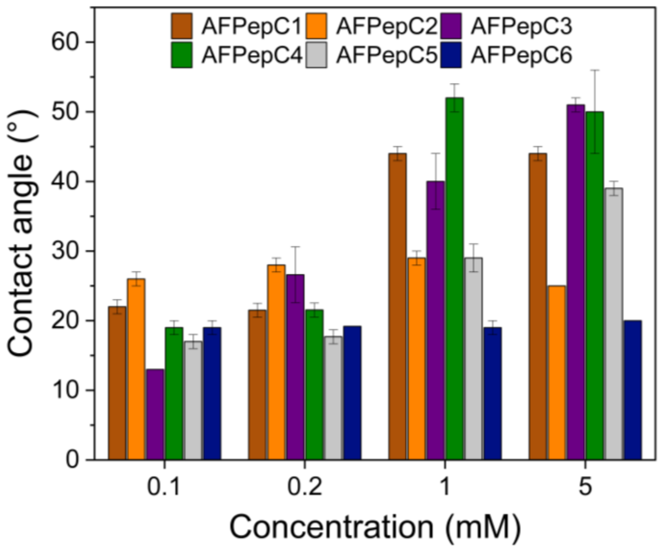

Figure S5. Water contact angle values for the peptides AFPepC1-AFPep C6. The standard deviation (SD) was calculated based on data from three independent surfaces conducted in three different areas.

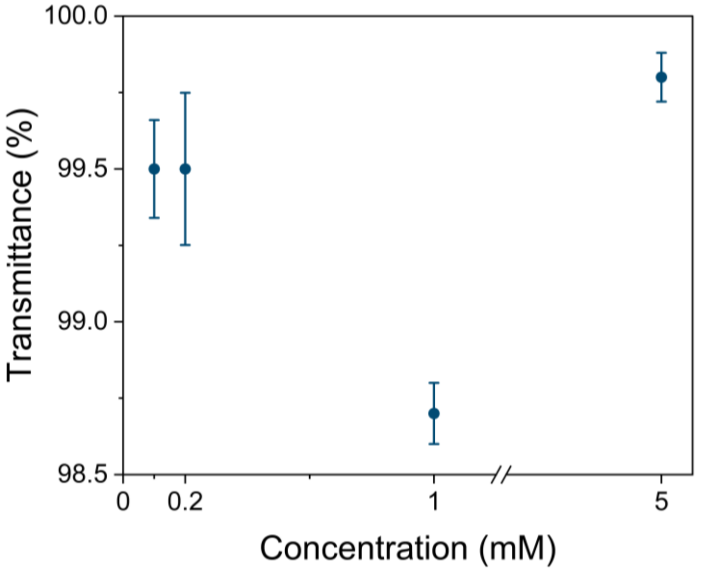

Figure S6. Transmittance of glass surfaces coated with AFPep1 at different concentrations (0.1 mM, 0.2 mM, 1 mM, and 5 mM). The SD was calculated based on data from three independent surfaces conducted in three different areas.

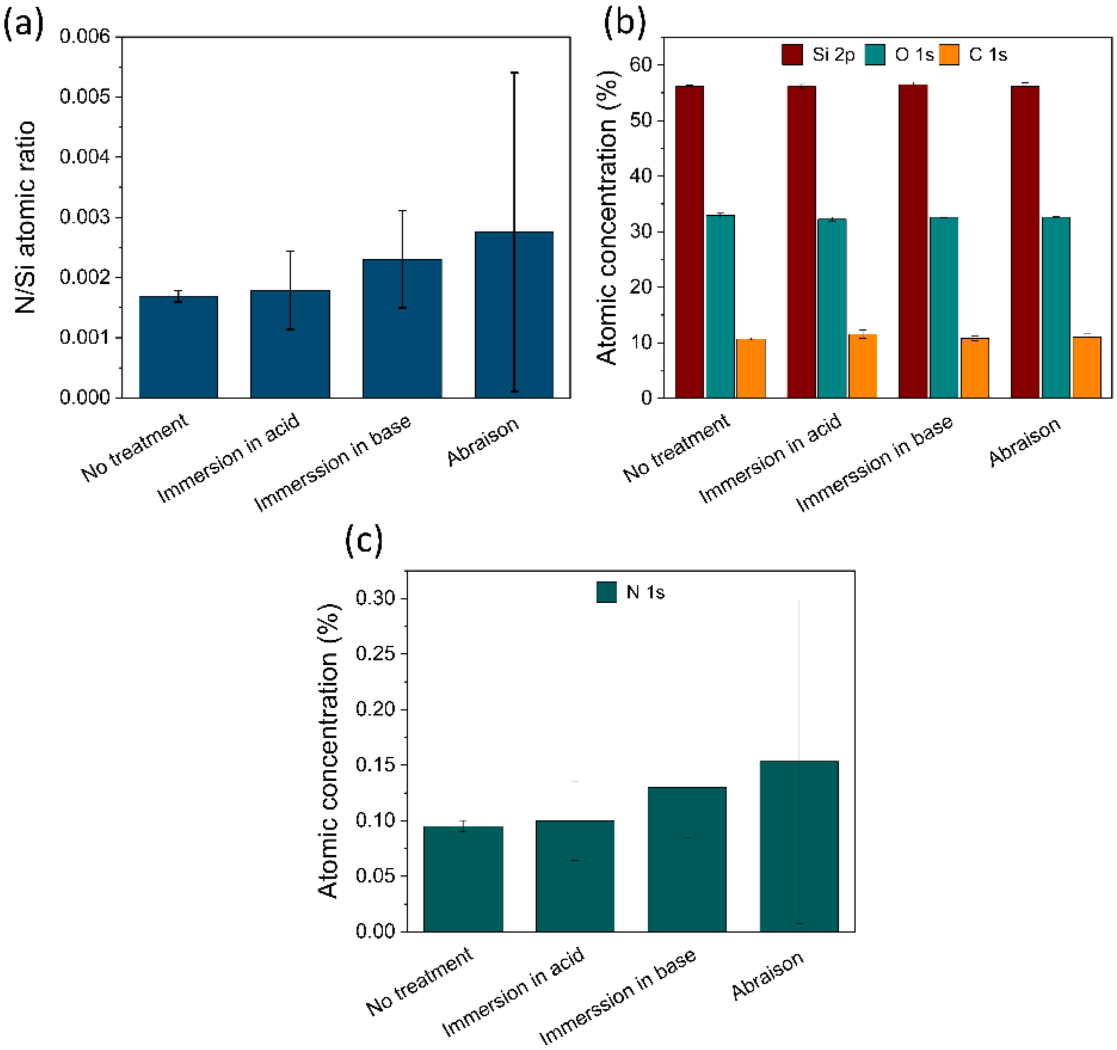

Figure S7. XPS analysis of peptide-coated surfaces before and after exposure to acidic, alkaline, or abrasion treatments. (a) N/Si atomic ratio, (b) Atomic percentages of Si, O, and C, and (c) Atomic percentages of N. The SD was calculated based on data from three independent surfaces.

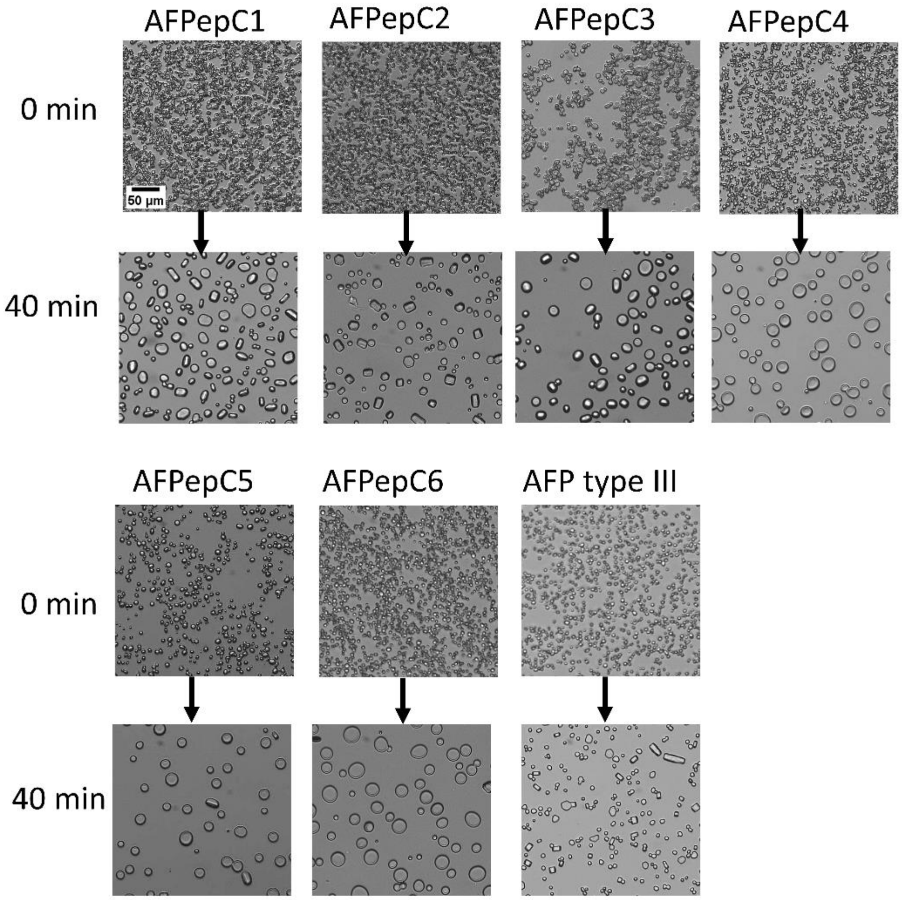

Figure S8. Microscopic images of ice crystals recrystallization at -8 ˚C in 45% sucrose solutions, including control peptides (AFPepC1-AFPepC6) at 2mM and AFP type III at 2 μM. The scale bar applies to all images.

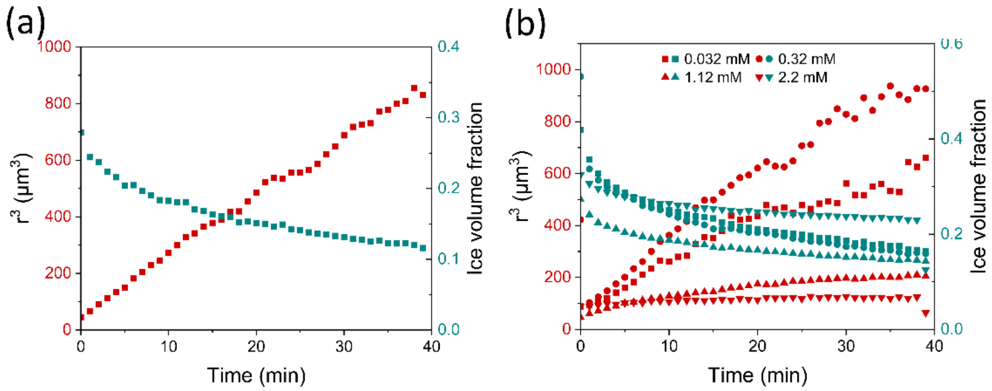

Figure S9. Representative results for a cubic mean radius of ice crystals and ice volume fraction during IRI experiment for (a) water and (b) AFPep1.

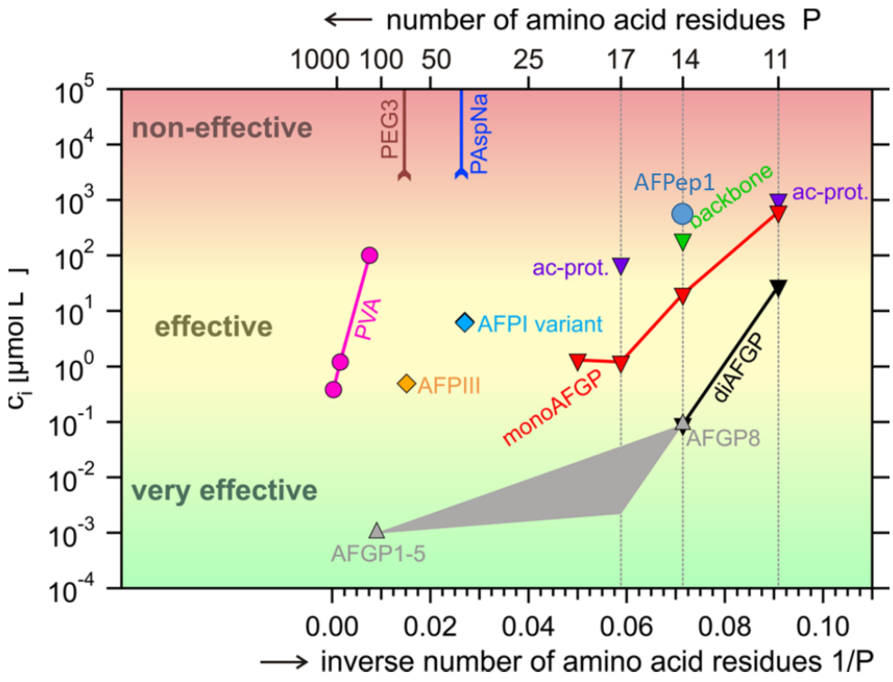

Figure S10. Ice recrystallization inhibition analysis effectivity. The position of AFPep1 among the effective IRI materials is indicated as a blue circle laid over the effectivity map taken by Budke et al.[1]

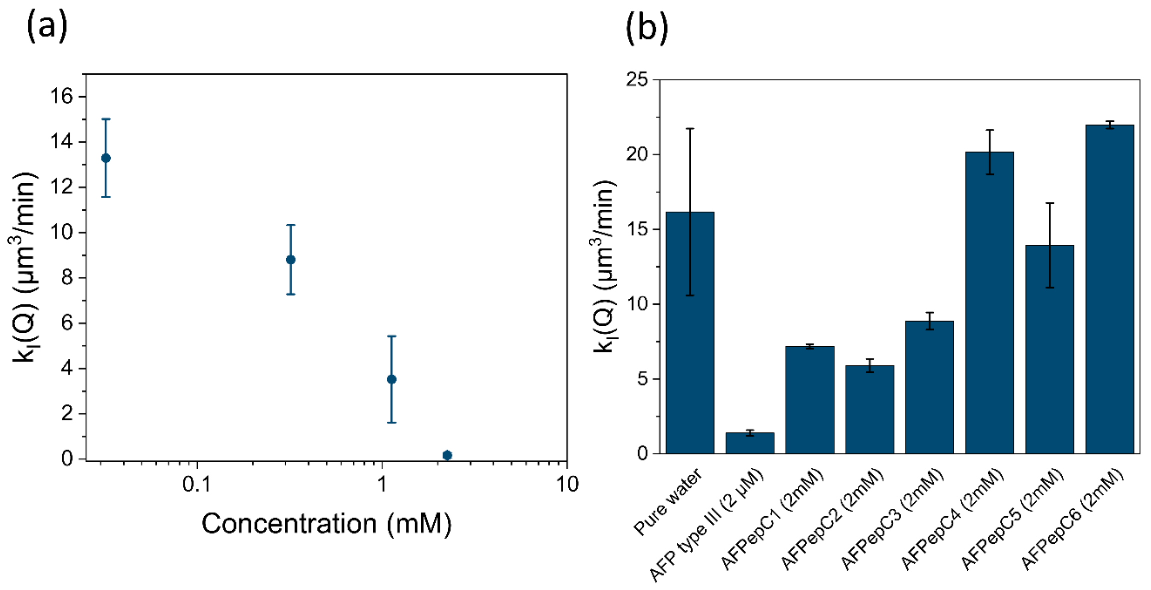

Figure S11. Ice recrystallization inhibition analysis. (a) The observed ice recrystallization rate constant for AFPep1 as a function of peptide concentration. (b) The observed ice recrystallization rate constant for sucrose 45 wt%, AFP type III at 2 µm, and control peptides (AFPepC1-AFPepC6) at 2 mM. The SD was calculated using data from a minimum of three independent samples.

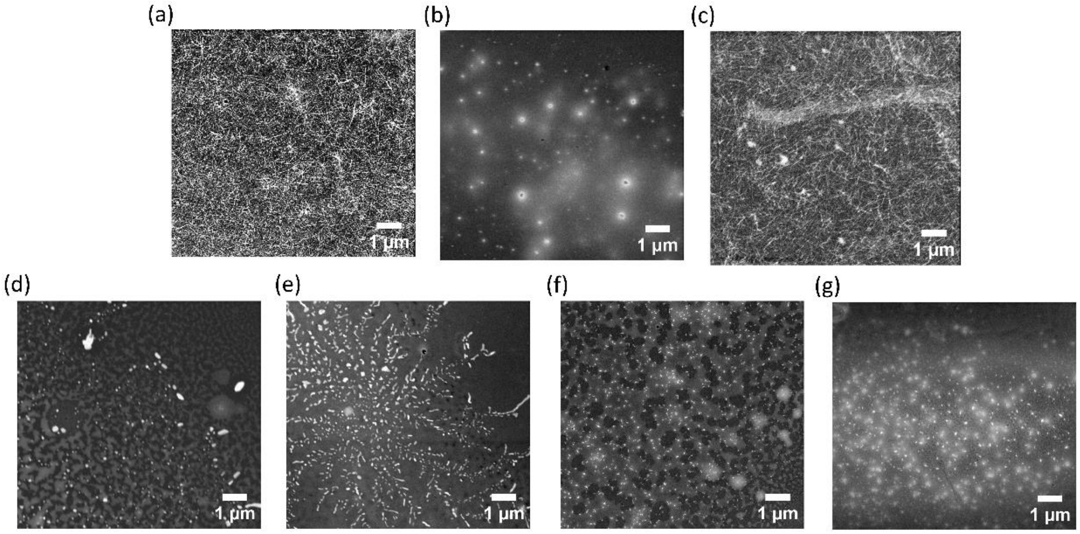

Figure S12. Representative HAADF-STEM images of (a) AFPep1, (b) AFPepC1, (c) AFPepC2, (d) AFPepC3, (e) AFPepC4, (f) AFPepC5, and (g) AFPepC6.

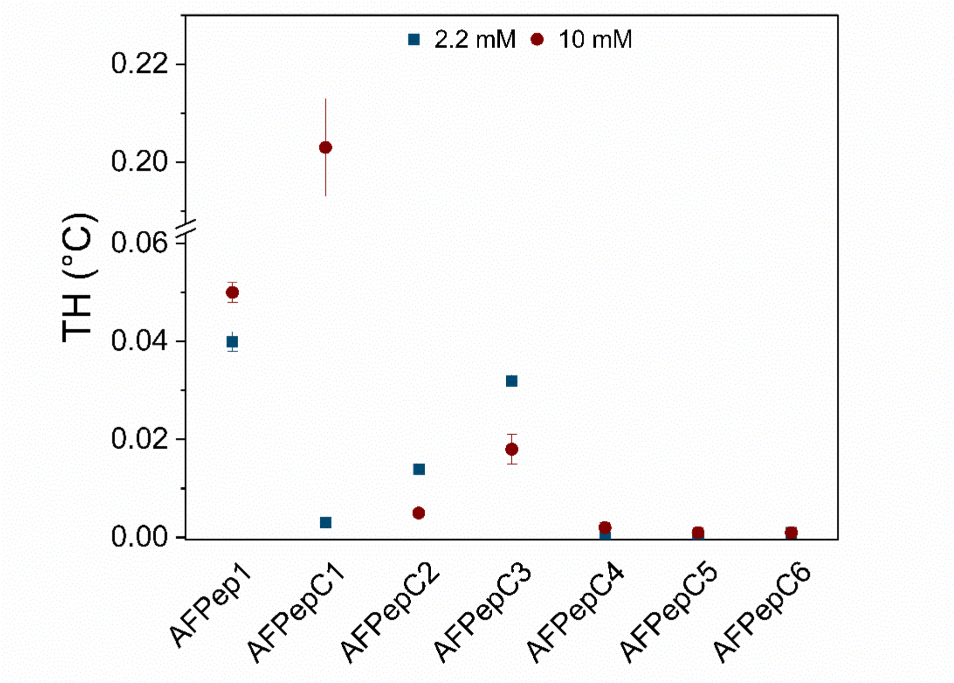

Figure S13. Thermal hysteresis of peptides AFPep1 and AFPepC1-AFPepC6 at concentrations of 2.2 mM and 10 mM. The SD was calculated using data from a minimum of three independent samples.

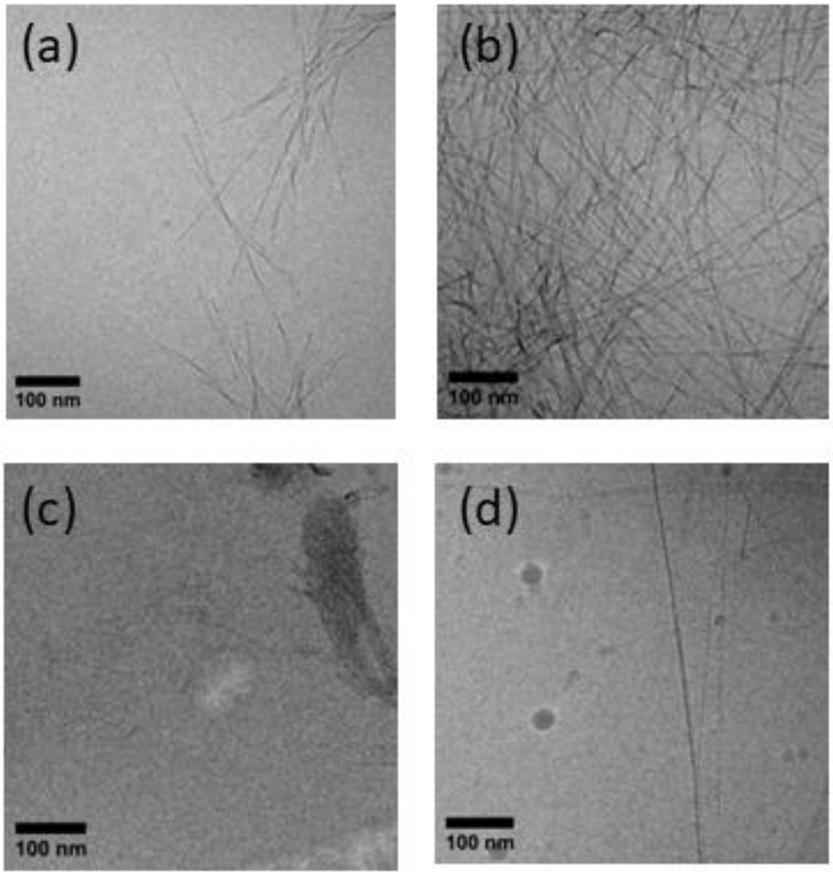

Figure S14. Cryo-TEM images of AFPep1 at concentrations of (a) 2.2 mM and (b) 10 mM, and AFPepC1 at concentrations of (c) 2.2 mM and (d) 10 mM.

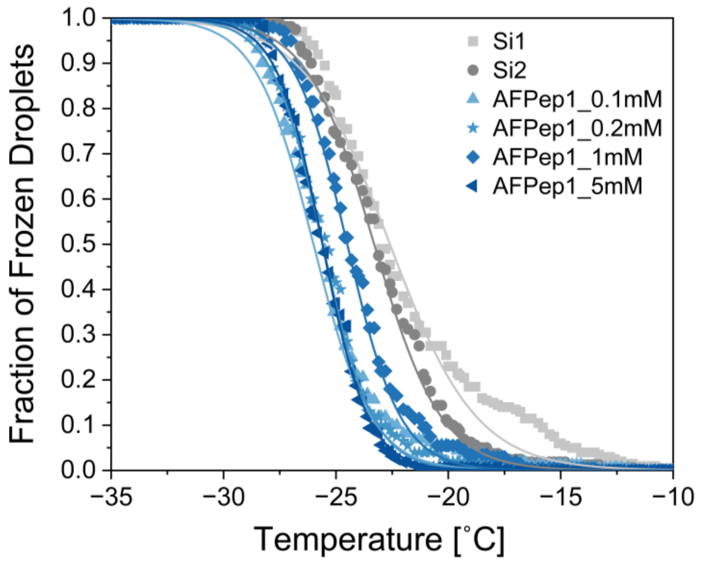

Figure S15. Fraction of frozen droplets at decreasing temperature for AFPep1.

Table S1. Surface roughness of bare silicon surfaces (Si1 and Si2) and peptide AFPep1 at different concentrations (5 mM, 1 mM, 0.2 mM, and 0.1 mM).

Surface	Roughness (nm)
**Si1**	0.8±0.3
**Si2**	2.8±0.9
**AFPep1 at 5 mM**	9.4±0.8
**AFPep1 at 1 mM**	7.7±0.9
**AFPep1 at 0.2 mM**	9±1
**AFPep1 at 0.1 mM**	6.2±0.9

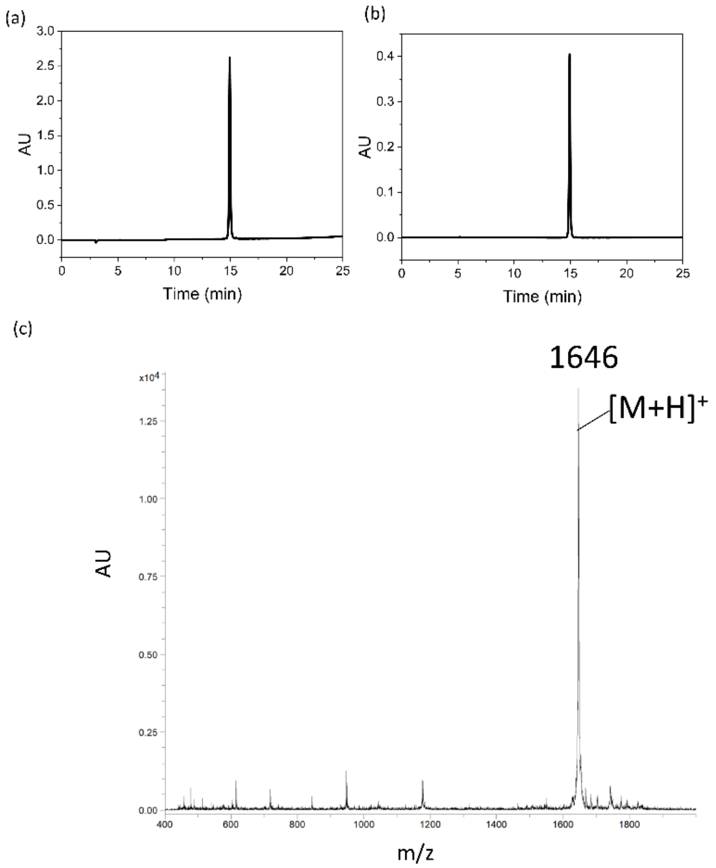

Figure S16. Analytical HPLC chromatograms of AFpep1 at wavelengths of 220 nm (a) and 280 nm (b), and mass spectrometry analysis (c).

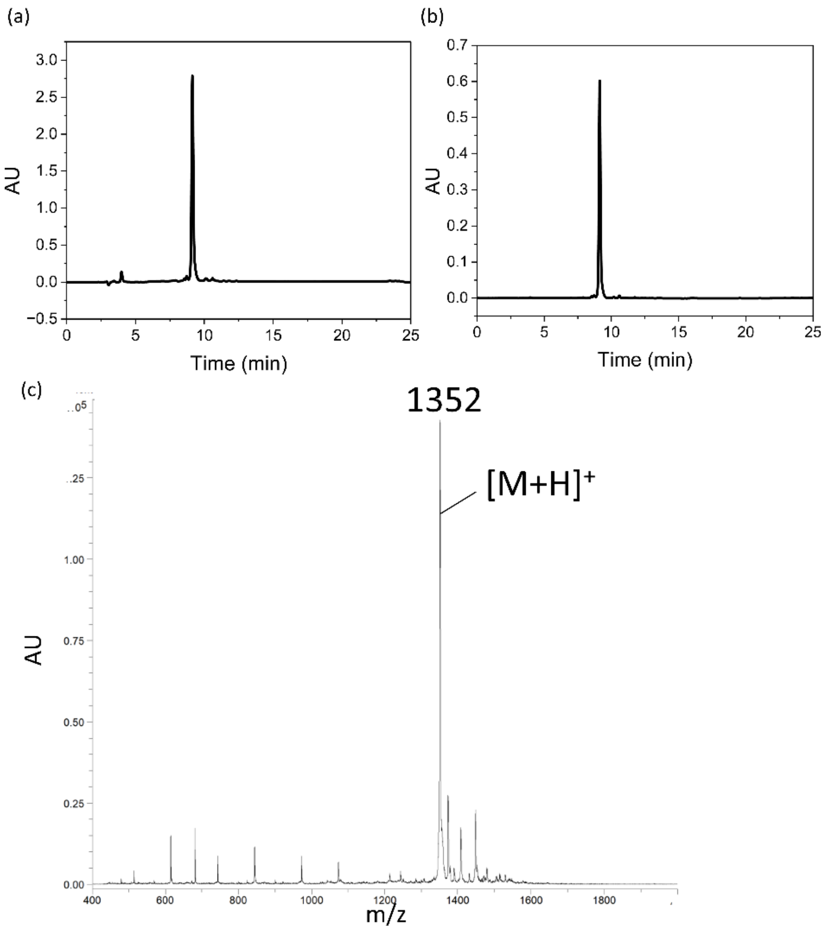

Figure S17. Analytical HPLC chromatograms of AFpepC1 at wavelengths of 220 nm (a) and 280 nm (b), and mass spectrometry analysis (c).

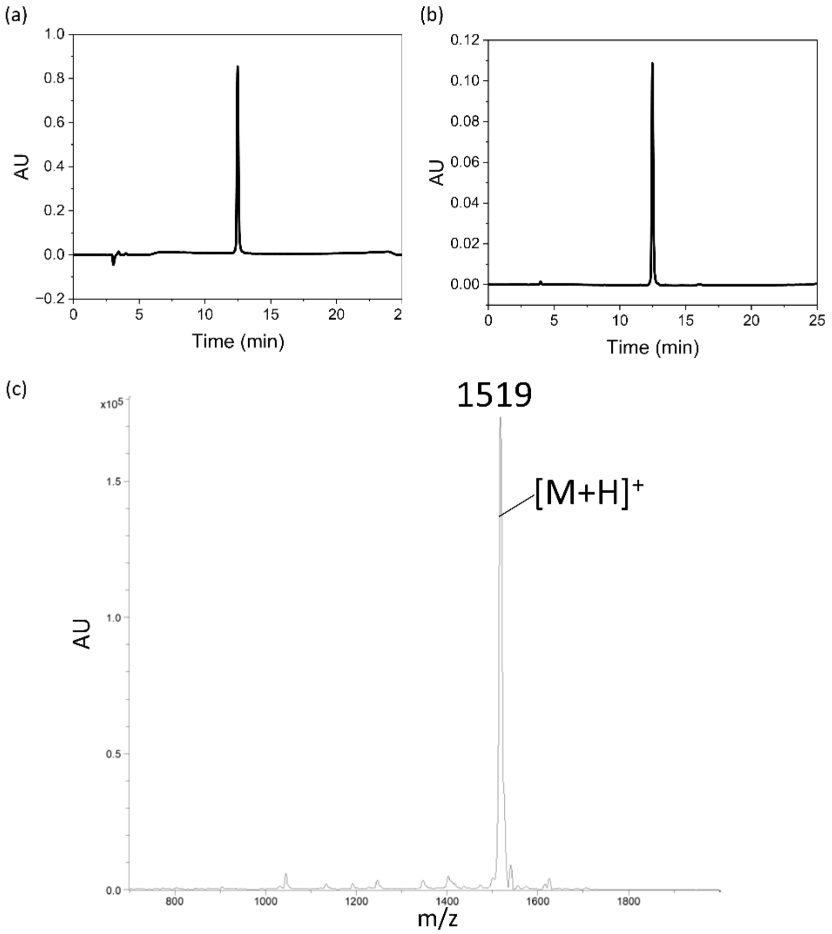

Figure S18. Analytical HPLC chromatograms of AFpepC2 at wavelengths of 220 nm (a) and 280 nm (b), and mass spectrometry analysis (c).

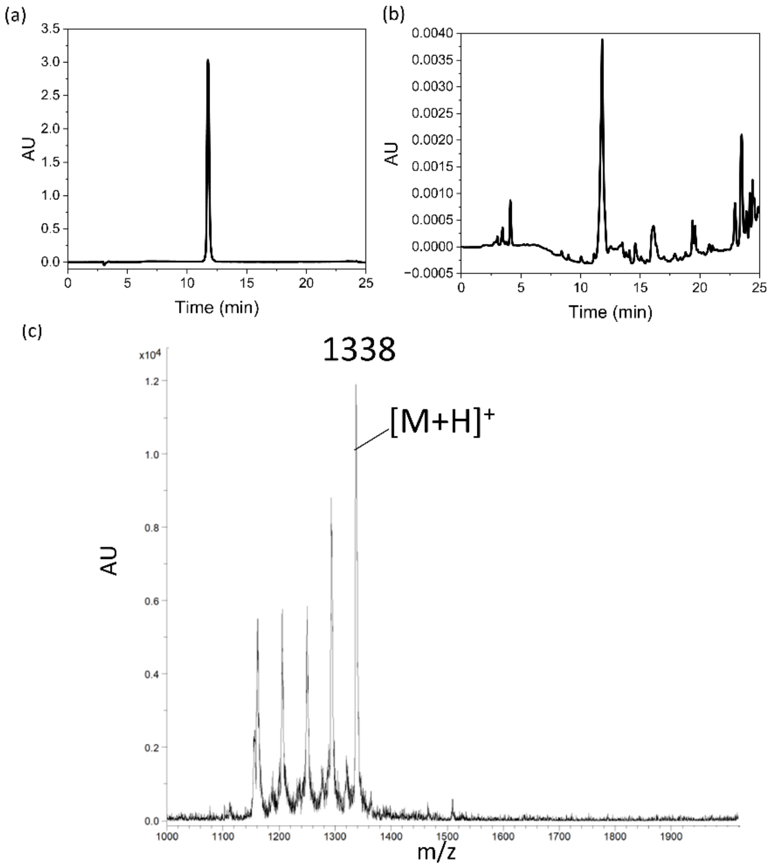

Figure S19. Analytical HPLC chromatograms of AFpepC3 at wavelengths of 220 nm (a) and 280 nm (b), and mass spectrometry analysis (c).

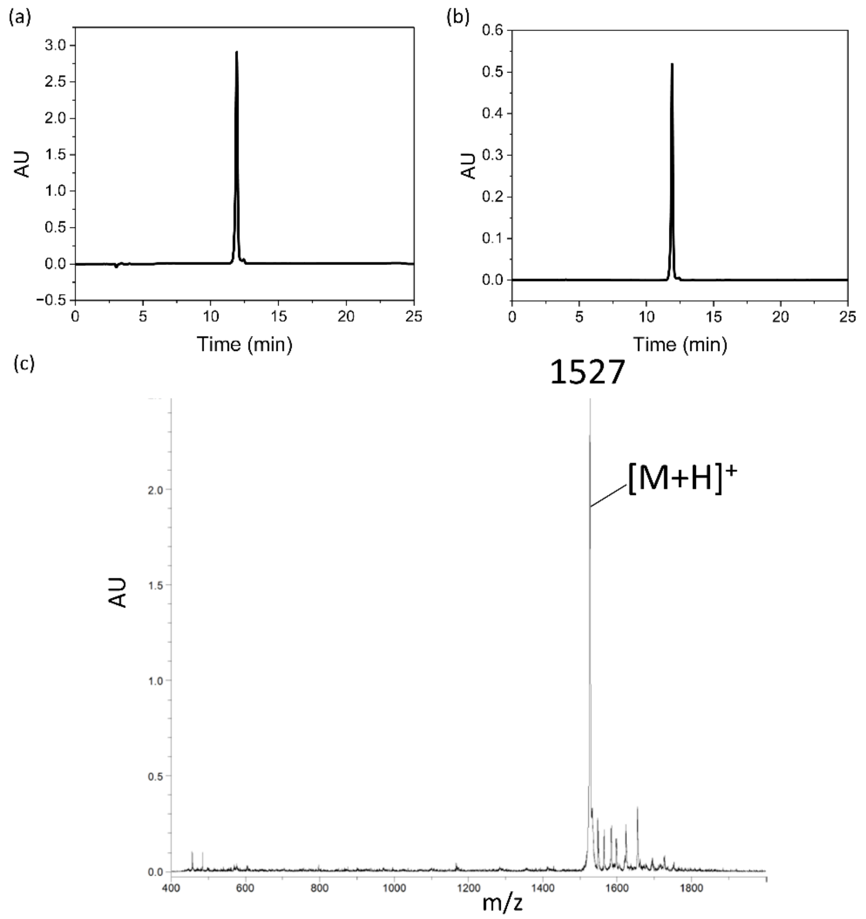

Figure S20. Analytical HPLC chromatograms of AFpepC4 at wavelengths of 220 nm (a) and 280 nm (b), and mass spectrometry analysis (c).

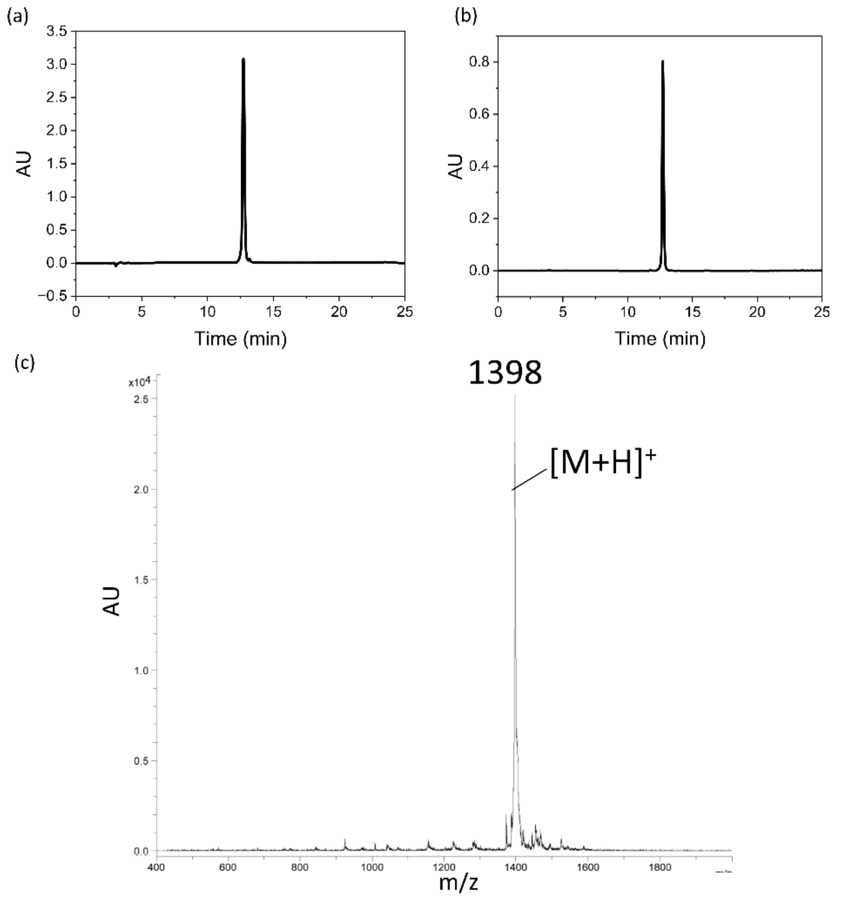

Figure S21. Analytical HPLC chromatograms of AFpepC5 at wavelengths of 220 nm (a) and 280 nm (b), and mass spectrometry analysis (c).

Movie S1- A movie showing crystal burst in a 10 mM AFPep1 solution.

REFERENCES

(1) Budke, C.; Dreyer, A.; Jaeger, J.; Gimpel, K.; Berkemeier, T.; Bonin, A. S.; Nagel, L.; Plattner, C.; Devries, A. L.; Sewald, N.; Koop, T. Quantitative Efficacy Classification of Ice Recrystallization Inhibition Agents. *Cryst Growth Des* **2014**, *14* (9), 4285–4294. https://doi.org/10.1021/cg5003308.
